# Indistinguishable Landscapes of Meiotic DNA Breaks in *rad50^+^* and *rad50S* Strains of Fission Yeast Revealed by a Novel *rad50^+^* Recombination Intermediate

**DOI:** 10.1371/journal.pgen.1000267

**Published:** 2008-11-21

**Authors:** Randy W. Hyppa, Gareth A. Cromie, Gerald R. Smith

**Affiliations:** Fred Hutchinson Cancer Research Center, Seattle, Washington, United States of America; The University of North Carolina at Chapel Hill, United States of America

## Abstract

The fission yeast *Schizosaccharomyces pombe* Rec12 protein, the homolog of Spo11 in other organisms, initiates meiotic recombination by creating DNA double-strand breaks (DSBs) and becoming covalently linked to the DNA ends of the break. This protein–DNA linkage has previously been detected only in mutants such as *rad50S* in which break repair is impeded and DSBs accumulate. In the budding yeast *Saccharomyces cerevisiae*, the DSB distribution in a *rad50S* mutant is markedly different from that in wild-type (*RAD50*) meiosis, and it was suggested that this might also be true for other organisms. Here, we show that we can detect Rec12-DNA linkages in *Sc. pombe rad50^+^* cells, which are proficient for DSB repair. In contrast to the results from *Sa. cerevisiae*, genome-wide microarray analysis of Rec12-DNA reveals indistinguishable meiotic DSB distributions in *rad50^+^* and *rad50S* strains of *Sc. pombe*. These results confirm our earlier findings describing the occurrence of widely spaced DSBs primarily in large intergenic regions of DNA and demonstrate the relevance and usefulness of fission yeast studies employing *rad50S*. We propose that the differential behavior of *rad50S* strains reflects a major difference in DSB regulation between the two species—specifically, the requirement for the Rad50-containing complex for DSB formation in budding yeast but not in fission yeast. Use of *rad50S* and related mutations may be a useful method for DSB analysis in other species.

## Author Summary

During meiosis, which creates haploid gametes from diploid cells, recombination between two homologous chromosomes increases genetic diversity and, in most organisms, is crucial for proper segregation of chromosomes into haploid nuclei. To better understand where recombination occurs and why it occurs there, we investigated in fission yeast the initiating step in recombination—formation of DNA double-strand breaks (DSBs). A genome-wide DSB map is crucial to understand how DNA sequence and chromatin structure affect DSB formation and may help answer these questions in other organisms, including humans. Mutants in which DSBs accumulate are particularly useful for determining the DSB distribution. As recently reported, however, in budding yeast the DSB distribution in one such widely used mutant, *rad50S*, differs markedly from that in a *dmc1* mutant, in which DSBs also accumulate and appear to have a more nearly wild-type distribution. We have detected in fission yeast a DNA–protein intermediate of recombination assumed to exist, but never before detected, in a recombination-proficient strain (*rad50^+^*). The distributions of this intermediate, and therefore those of DSBs, in *rad50^+^* and *rad50S* strains are indistinguishable. *rad50S*-like mutations may also accurately reflect the wild-type DSB distribution in other species and may be particularly useful in species lacking Dmc1 orthologs.

## Introduction

Sexual reproduction involves the fusion of two gametes to create diploid offspring with equal genetic contributions from each parent. To maintain the proper chromosome number (ploidy), it is therefore necessary for the gametes to be haploid. This is achieved via meiosis, where a single round of DNA replication is followed by two nuclear divisions: in the first division, homologous chromosomes (homologs) separate from each other (Meiosis I), followed in the second division by the separation of sister chromatids (Meiosis II). Meiotic recombination, a highly conserved feature of meiosis, creates between the homologs a physical connection that is necessary in most species for proper homolog segregation during Meiosis I.

Before the first meiotic division, homologs become aligned and then intimately synapsed [1]. During this time meiotic recombination is initiated by DNA double-strand breaks (DSBs), introduced by Spo11 in the budding yeast *Saccharomyces cerevisiae* or its ortholog Rec12 in the fission yeast *Schizosaccharomyces pombe* [2]. The DNA ends undergo 5′ to 3′ resection, producing 3′ single-stranded (ss) ends capable of invading intact homologous DNA, with the invaded duplex serving as the template for new DNA synthesis [3]. Resolution of the joint DNA molecules can result in a reciprocal exchange of genetic information, called a crossover, which aids proper homolog segregation at Meiosis I. This exchange of genetic material is also beneficial in that it enhances the genetic diversity of the progeny.

As a type II topoisomerase-like protein, Spo11 (or Rec12) breaks phosphodiester bonds in the two DNA strands and becomes covalently bound to each 5′ DNA end of the DSB [4],[5]. This DNA-protein linkage enables determination of where DSBs are made, by chromatin immunoprecipitation (ChIP) of Spo11 or Rec12 and assay of the attached DNA, *e.g.* by tiling microarray hybridization. In wild-type cells the Spo11 protein is removed from the DNA end by endonuclease action before strand resection occurs [6]. In *rad50S* mutants, the bound Spo11 or Rec12 protein is not removed from the DNA ends, repair of the DSB by recombination is blocked, and the protein-bound DSBs accumulate [2]. This has facilitated genome-wide analysis of the DSB distribution in both fission and budding yeast strains with the *rad50S* mutation. These comprehensive DSB maps revealed in both organisms regions of DNA within which DSBs are made at high frequency, called DSB hotspots [5],^7^.

Two recent studies [8],[9] described a new technique for genome-wide mapping of DSBs that cast doubt on earlier results where a *rad50S* mutant was used. It had previously been observed that DSBs in *S. cerevisiae* strains with a *rad50S* mutation (or mutations in other genes with similar phenotypes, such as *sae2Δ* and *mre11S*) did not show in many regions of the genome the same DSB pattern as that in *dmc1 Δ* mutants [10]. In addition, the overall genetic map of recombination (crossovers) did not agree in certain intervals with the frequency of DSBs determined in *rad50S* strains [11]. This led Buhler et al. [8] and Blitzblau et al. [9] to develop a method that enriched for regions of ss DNA formed in a *dmc1* mutant; *dmc1* mutants lack a protein important for strand exchange, and DSBs with resected ends accumulate in these mutants. The enriched ss DNA was hybridized to a genome-wide tiling microarray of oligonucleotides to identify sites of DSBs. The results showed, in many but not all regions of the genome, a clear under-representation of DSB hotspots in *rad50S*-like mutants compared to the distributions in the wild type or *dmc1Δ* mutant, which appear similar by Southern blot analysis. Specifically, the intensity of breakage at some, but not all, DSB hotspots was greatly reduced in the *rad50S*-like mutants compared to that in the wild type or *dmc1Δ* mutant. The validity of DSB maps created with *rad50S* mutants, not only in *S. cerevisiae* but in other organisms as well, is therefore under new scrutiny.

Our lab has reported that DSB hotspots in *S. pombe* are preferentially located in large intergenic regions and are widely-spaced – on average there are about 65 kb between hotspots – but these experiments were done with *rad50S* mutants [5],[12]. We wanted to know if a wild-type (*rad50^+^*) strain has a DSB map similar to that seen in *rad50S* mutants. ChIP experiments to detect the Spo11-DNA covalently linked intermediates in *RAD50* strains of budding yeast have not been successful [13],[14; M. Lichten, personal communication], apparently due to the short life-span of the hypothesized Spo11-DNA complexes. By contrast, in fission yeast we were able to detect and analyze the wild-type (*rad50^+^*) Rec12-DNA complexes. To our knowledge, this is the first time that this protein-DNA intermediate has been detected in recombination-proficient cells. We report here that the locations of DSBs, measured as Rec12-DNA linkages, across the genome in *S. pombe rad50^+^* meiosis are indistinguishable from those in *rad50S* strains, although the intensities are lower, as expected due to ongoing DSB repair in *rad50^+^* strains. Therefore, conclusions from our earlier studies using the *rad50S* mutation are still valid: in particular, DSBs are separated by large distances and are preferentially located in large intergenic regions [5],[12]. However, the genetic recombination maps (crossover distributions) and physical maps (DSB distributions in *rad50^+^* and *rad50S* strains) display non-congruence in *S. pombe*. We discuss the significance of these observations for studies of meiotic recombination in *S. pombe* and in other species, including humans.

## Results

### Meiotic DSB Hotspots Mapped by Southern Blots Are the Same in *rad50^+^* and *rad50S* Strains

**Figure 1:**
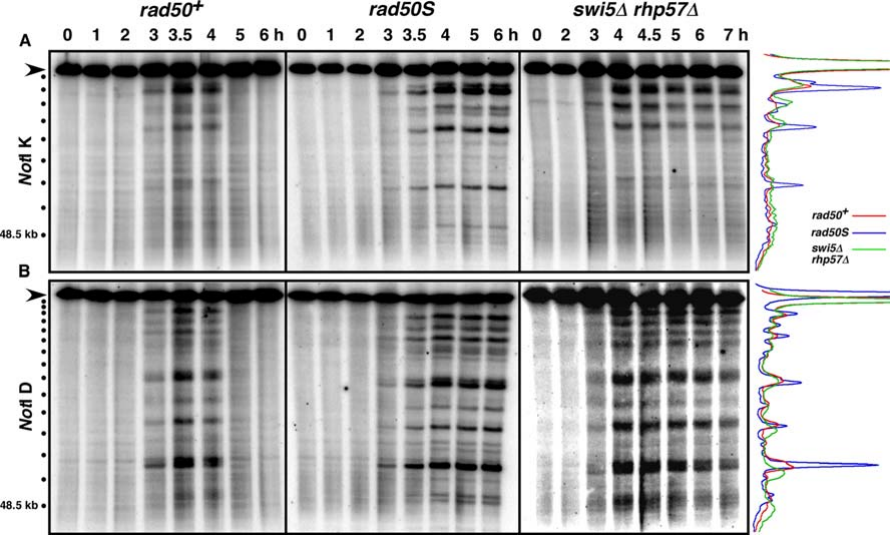
Southern Blots Reveal Indistinguishable DSB Hotspots in *rad50^+^* and *rad50S* Strains. DNA from meiotically induced strains was digested with *Not*I, and the fragments separated by pulsed-field gel electrophoresis. Inductions were performed concurrently with *rad50^+^* (GP1979; left), *rad50S* (GP3718; middle), and *swi5Δ rhp57Δ* (GP6658; right) strains. (A) The blots were probed on the left end of the 480 kb *Not*I restriction fragment K (black arrowhead) of chromosome I. (B) The same blots were probed on the left end of the 1.2 Mb *Not*I restriction fragment D (black arrowhead) of chromosome I. On the left are size markers, 48.5 kb phage lambda DNA (lowest bullet) and its concatemers. On the right are lane traces of the time of maximal DSBs [3.5 h *rad50^+^* (blue) and 5 h *rad50S* (red), and 4.5 h *swi5Δ rhp57Δ* (green)] for each probing. Similar results were obtained in an independent induction of the *rad50^+^* strain (Figure S2).

We began our comparison of the DSB distributions in *rad50^+^* and *rad50S* strains by assaying DSBs using standard Southern blots. *rad50^+^* and *rad50S* strains were meiotically induced (Figure S1), and the DNA was extracted and digested with *Not*I restriction enzyme to generate large DNA fragments, which were separated by pulsed-field gel electrophoresis. Previous Southern blot analyses of DNA from *rad50^+^* and *rad50S* strains revealed the same meiotic DSB pattern on the 0.5 Mb *Not*I restriction fragment J, which includes the well-characterized DSB hotspot *mbs1* [5],[12],[15]. Two additional *Not*I fragments were probed to strengthen this observation; these analyses were of the 0.5 Mb *Not*I fragment K (Figure 1A and S2A) and the 1.2 Mb *Not*I fragment D (Figure 1B and S2B). These results revealed that *rad50^+^* strains have on each fragment multiple DSB sites at the same locations as those from a *rad50S* strain. As expected, in almost all cases the maximal level of the transient DSBs in the *rad50^+^* strain was less than that in the *rad50S* strain, in which DSBs accumulate. At each hotspot site on these *Not*I fragments in a *rad50^+^* strain there is a hotspot in the *rad50S* strain, and vice versa.

We next compared DSB sites in a repair-deficient mutant other than *rad50S* in which DSBs accumulate. During meiotic recombination in *S. pombe* there are two mediator complexes that assist the strand exchange protein Rhp51 in strand invasion: Swi5-Sfr1 and Rhp55-Rhp57 [16],[17]. Mutants lacking either complex show reduced recombination and delayed DSB repair, and strains with a mutation in both complexes display recombination defects and spore viability as severe as an *rhp51* null mutant [16],[18] but slightly better growth and meiotic induction than an *rhp51* null mutant (RWH, unpublished data). Thus, the double *swi5Δ rhp57Δ* mutant is an ideal candidate for assaying defective DSB repair at a stage later than the *rad50S* repair defect, allowing for DSB accumulation in a non-*rad50S* strain. Southern blot analysis of *Not*I fragments K (Figure 1A), D (Figure 1B), and J (data not shown) from the *swi5Δ rhp57Δ* mutant revealed a DSB pattern similar to those seen in *rad50^+^* and *rad50S* strains, except that the broken DNA persisted in *rad50S* and *swi5Δ rhp57Δ* mutants but was repaired in wild type. Thus, by Southern blot analysis in the *rad50S* mutant there is no lack of DSB sites that are present in other mutants, unlike the situation in *S. cerevisiae*, as noted above [8],[9].

### ChIP Enrichment Shows Transient Rec12-DNA Covalent Linkage in *rad50^+^* Strains

**Figure 2:**
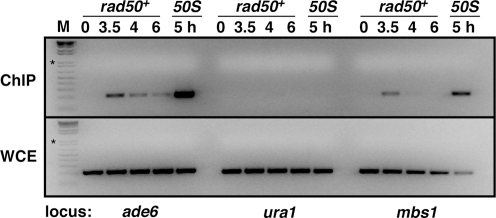
Rec12-DNA Covalent Linkages Are Readily Detectable in *rad50^+^* Strains. Chromatin was prepared from meiotically induced *rad50^+^* (GP6232) and *rad50S* (GP6203) strains bearing FLAG-tagged Rec12 and immunoprecipitated with anti-FLAG antibody. Enrichment for Rec12-linked DNA was assayed by PCR at two DSB hotspots (*ade6-3049* and *mbs1*) and one coldspot (*ura1*); template for the PCR was immunoprecipitate (ChIP) or whole cell extract (WCE). The maximal PCR signal was at 3.5 h in the *rad50^+^* strain, in agreement with the time of maximal DSBs (Figure 1). In addition, there is little or no signal before meiotic induction (0 h); the signal decreases as meiosis progresses, indicating the initial step of DSB repair. The 5 h *rad50S* DNA shows a much stronger enrichment (ratio of PCR product from the ChIP to that from the WCE) as a result of Rec12 not being removed from the DNA ends in that strain. Neither strain showed any meiotic enrichment of Rec12-DNA linkages in the DSB coldspot in *ura1*. On the left are size markers (1 Kb Plus DNA Ladder; Invitrogen); asterisk, 1 kb.

Since Rec12 becomes covalently bound to the DNA ends at a meiotic DSB [5], ChIP of epitope-tagged Rec12 protein without exogenous cross-linking can identify the genomic loci where DSBs occur. Previous ChIP analysis of FLAG-tagged Rec12 in *rad50S* meiosis showed that DSB hotspots assayed by locus-specific PCR gave a meiosis-specific signal dependent on Rec12 (*i.e.*, DSB formation), while DSB coldspots gave no detectable signal [5]. We wanted to know if it was possible to repeat this analysis in a *rad50^+^* meiosis, or if Rec12 was removed from the DNA too quickly to be detected, as appears to be the case in budding yeast [13],[14]. PCR analysis of two prominent DSB hotspots, *ade6-3049* on chromosome III [19] and *mbs1* on chromosome I, revealed that DNA isolated 3.5 h after induction of meiosis was considerably enriched by ChIP when compared to 0 h (uninduced) DNA, based on the relative abundance of PCR products. This was true for DNA from a *rad50^+^* strain as well as from a *rad50S* strain, though as expected enrichment was lower in the *rad50^+^* strain due to ongoing repair of the DSBs (Figure 2). There was no detectable enrichment at the DSB coldspot *ura1* (Figure 2C and [5]). In addition, the enrichment at the hotspots in *rad50^+^* was transient: very little signal was detected at 0 h (before DSB formation) or at 6 h after meiotic induction (after DSB repair). The maximal signal was at 3.5 h, which is about the time of maximal DSBs detectable by Southern blots in *rad50^+^* strains (Figure 1 and [5]). This contrasts with a *rad50S* meiosis, where the PCR assay detects high DNA enrichment at least to 6 h after meiotic induction [5], a reflection of Rec12 remaining bound and the DSBs not being repaired in *rad50S* meiosis.

As an additional test for Rec12-DNA linkages in *rad50^+^* strains, we treated meiotic extracts with a protease (or not, as a control) and extracted the material with phenol. Protein-linked DNA is removed from the aqueous phase by phenol extraction [20]. A significant fraction of the DNA at the *mbs1* and *ade6-3049* DSB hotspots was removed by phenol extraction, as expected for DNA covalently linked to Rec12 protein, unless the extracted material was treated with a protease before extraction. This was true for material from both *rad50^+^* and *rad50S* strains (Figure S3), and contrasts sharply with results from *S. cerevisiae*, in which no detectable DNA is removed by phenol extraction in *RAD50* strains [20]. Our results show that a significant fraction of the DNA at DSB hotspots in *S. pombe rad50^+^* strains remains linked to a protein, likely Rec12.

### Genome-Wide Distribution of Rec12-DNA ChIP in *rad50^+^* Strains Is Coincident with That in *rad50S* Strains

To extend these observations to the entire genome, we used a genome-wide microarray analysis similar to our previous analysis with *rad50S* strains [5]. We prepared Rec12-DNA samples from immunoprecipitated (IP) chromatin and from whole-cell extracts (WCE) prepared at 0 and 3.5 h in *rad50^+^* meiosis and at 0 and 5 h in *rad50S* meiosis. These samples were amplified, differentially labeled, and hybridized to a tiling oligonucleotide microarray (∼44,000 60-mers, “probes,” spaced approximately every 290 bp across ∼12.5 Mb of the non-repetitive *S. pombe* genome). The relative frequency of Rec12-DNA linkage at each probe position was measured as the median-normalized ratio of IP signal to WCE signal. The 0 h data [log (IP/WCE) values] were normally distributed, as expected for random background data (Figure S4). In contrast, a distinct subset of probes in both the 3.5 h *rad50^+^* and 5 h *rad50S* data showed elevated non-normal ratios, reflecting genuine enrichment over background. The analysis below is focused on these enriched values.

**Figure 3:**
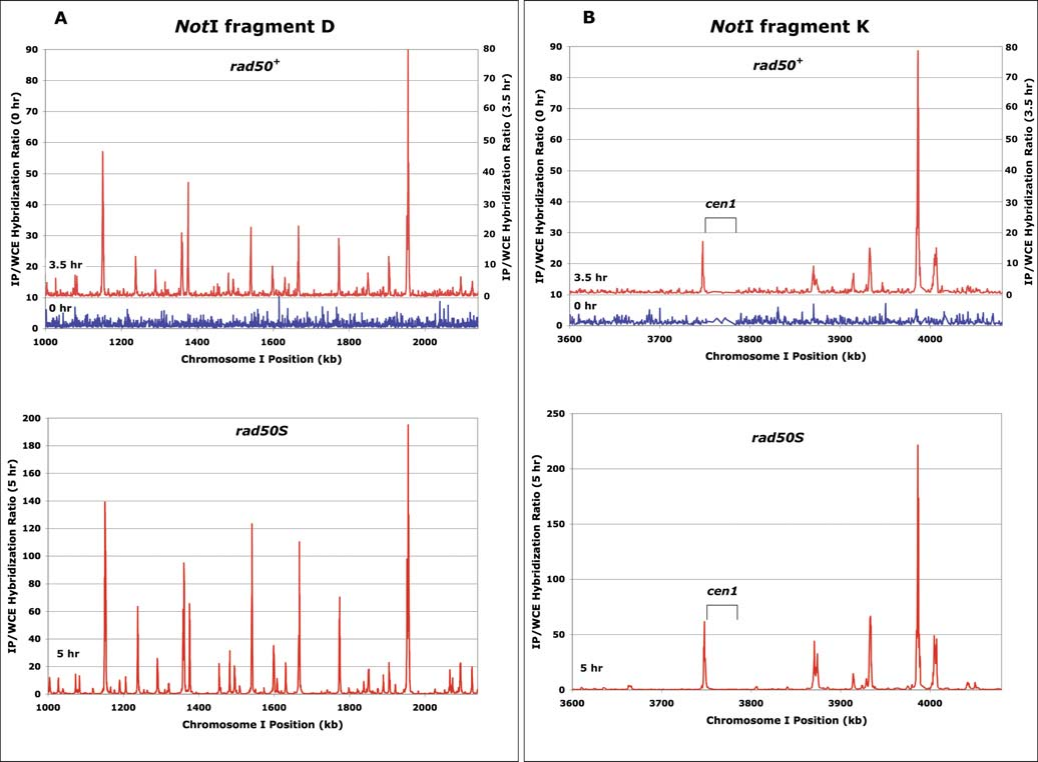
Microarray Analysis Reveals Indistinguishable DSB Hotspot Patterns in *rad50^+^* and *rad50S* Strains. Line graphs represent the median-normalized Rec12 IP/WCE ratios from the (A) *Not*I D and (B) *Not*I K restriction fragments, which were examined by Southern blot analysis (Figure 1). Peaks of IP enrichment in the *rad50^+^* (top, GP6232) and *rad50S* (bottom, GP6203) strains map to the same locations, with the *rad50S* strain showing, as expected, a higher level of enrichment due to accumulation of unrepaired DSBs. The data, from Dataset S2, are neither filtered for spurious values nor smoothed.

The data show that the sites of Rec12-DNA linkage, and hence the sites of meiotic DSBs, in a *rad50^+^* meiosis almost completely coincide with those in a *rad50S* meiosis. The genomic intervals of *Not*I fragment K and *Not*I fragment D, analyzed for DSBs by Southern blot analysis (Figure 1A and B), are compared by microarray analysis in Figure 3. There are no significant peaks of Rec12-DNA linkage in *rad50^+^* that are not also in *rad50S*; this correspondence is true genome-wide, as well (Figure S5). This result is dramatically different from that observed in *S. cerevisiae*, where multiple genomic regions show many more DSB hotspots in *RAD50* (*dmc1Δ*) meiosis than in *rad50S* meiosis, as measured by the enrichment for accumulated ss DNA ends [8],[9]. Within these regions, at many DSB hotspots seen in *RAD50* (*dmc1Δ*) the level of breakage in *rad50S* falls below the authors' definition of a hotspot. We note in particular that the DSB patterns surrounding the centromeres in *rad50S* and *rad50^+^* strains are indistinguishable in *S. pombe* (Figures 3 and S5) but markedly different in *S. cerevisiae* [8],[9].

**Figure 4:**
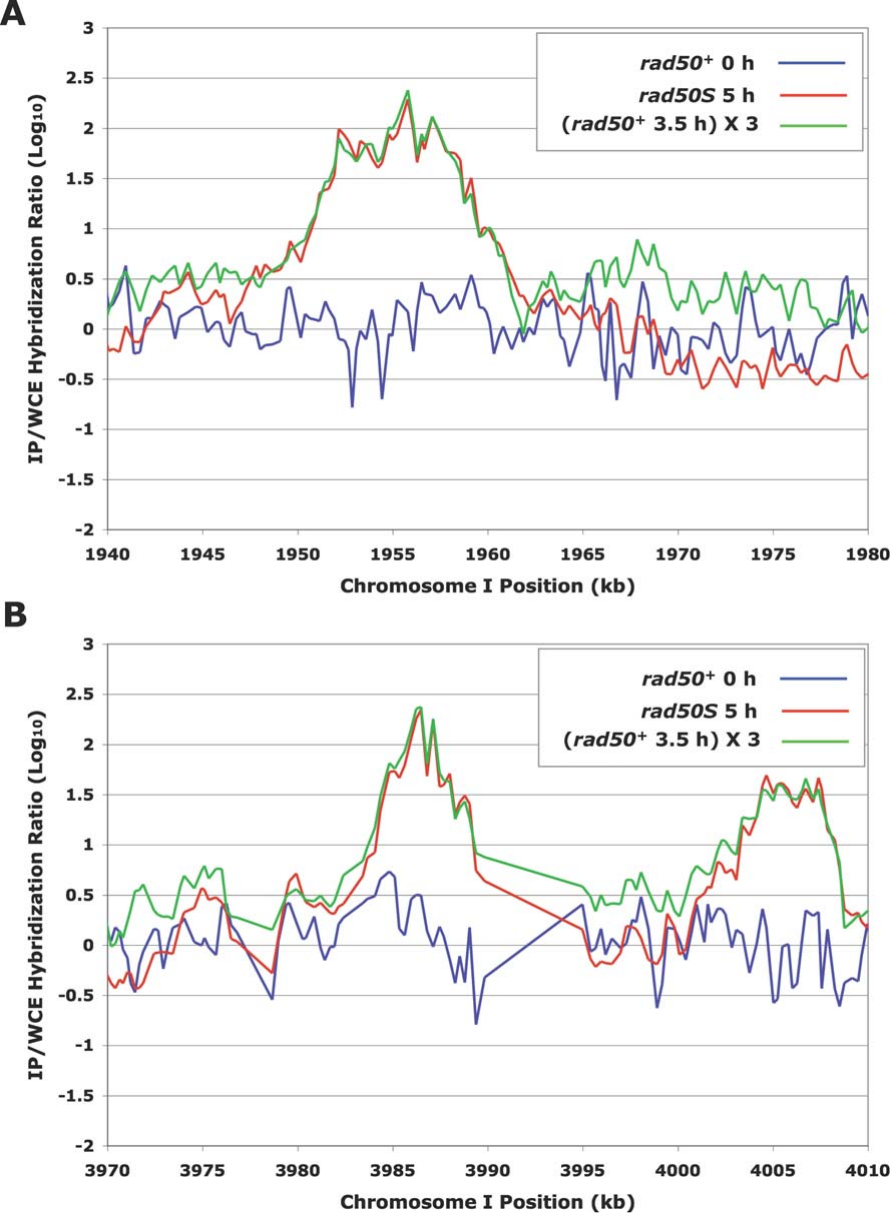
Enrichment Ratios at DSB Hotspots are Highly Correlated in *rad50^+^* and *rad50S* Strains. Line graphs represent the median-normalized Rec12 IP/WCE ratios at hotspot sites from (A) *Not*I D and (B) *Not*I K restriction fragments using Dataset S2. At probes showing IP enrichment (peaks), IP/WCE ratios are closely correlated between the *rad50^+^* (GP6232) and *rad50S* (GP6203) data, with ratios ∼3-fold higher in the *rad50S* data.

**Figure 5:**
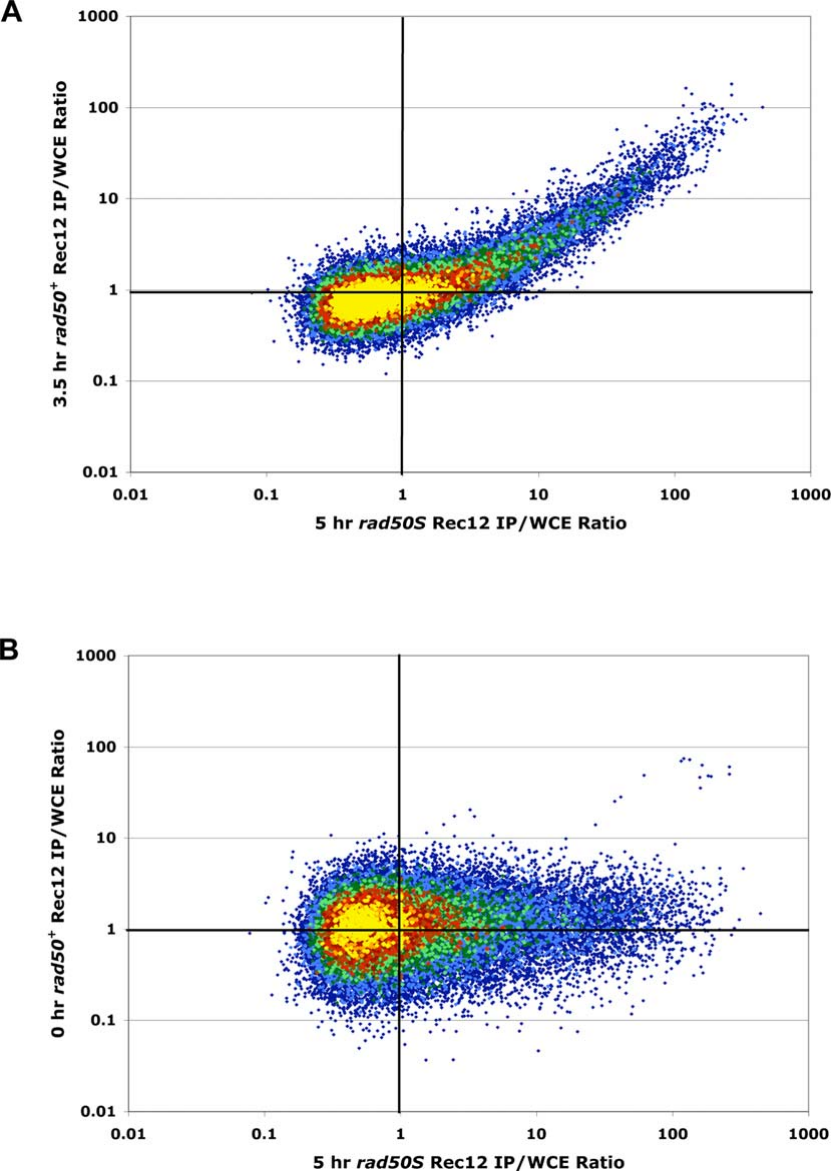
Essentially All DSB Hotspots Detected in *rad50^+^* Are Also DSB Hotspots in *rad50S*. The Rec12 IP/WCE ratio of each probe in the *rad50^+^* microarray hybridization is plotted against the IP/WCE ratio of the same probe in the 5 h *rad50S* microarray hybridization, using Dataset S2. Color indicates density of plotted points with yellow highest and dark blue lowest (see Figure S6). The plots are on a log scale. (A) The 3.5 h *rad50^+^* IP/WCE ratios are positively correlated with those of *rad50S*. Essentially all probes showing enrichment of the *rad50^+^* DNA by Rec12 IP also show enrichment in the *rad50S* DNA and *vice versa*. (B) The uninduced (0 h) *rad50^+^* IP/WCE ratios show no correlation with those of *rad50S*, as expected for uninduced background signals. Similar results were obtained in an independent induction of each strain (Dataset S1; Figure S6). In this experiment, but not the other (Figure S6), a small number of probes aberrantly displayed apparent enrichment in the 0 h *rad50^+^* data (B, top right).

Closer examination of one hotspot from each of *Not*I fragments K and D revealed that the shape of the enrichment peaks, considering non-background probes, was essentially identical for the *rad50^+^* and *rad50S* datasets, but with ∼3-fold less enrichment in the *rad50^+^* experiment (Figure 4). This 3-fold difference is consistent with comparisons of maximal meiotic DSB frequencies in *rad50^+^* and *rad50S* strains by Southern blot analysis [5; unpublished data] and appears to hold true genome-wide (Figures 5A, S6A and S7A). The matching peak shapes indicate that the Rec12-DNA shear sizes, DSB positions, and relative DSB intensities are nearly identical in the *rad50^+^* and *rad50S* experiments. This result rules out the possibility that the Rec12-DNA species detected by microarray hybridization in the *rad50^+^* experiment involves a significantly different length of DNA than that in the *rad50S* experiment. More specifically, we can discount the Rec12-DNA species in the *rad50^+^* experiment being a short Rec12-oligonucleotide released after DSB end-processing [6], rather than the Rec12-DNA intermediate first formed by Rec12 and accumulating in the *rad50S* background. In fact, such a short Rec12-oligonucleotide would not be amplified and hybridized in the procedure used here. As expected, the lengths of the two strands of DNA extending from one side of the DSBs at one hotspot to a common restriction site were similar (Figure S8), suggesting that at least some of the 5′ ends remain full length (*i.e.*, attached to Rec12).

### All Detectable Meiotic DSB Hotspots in *rad50^+^* Are Also Hotspots in *rad50S*

We analyzed our genome-wide data on a probe-by-probe basis to determine if hotspots of DSBs were at the same positions in both *rad50^+^* and *rad50S*; *i.e.*, are the probes with high IP/WCE ratios in *rad50^+^* also high in *rad50S*? For each of the ∼44,000 probes on the microarray, the IP/WCE ratio from the 3.5 h *rad50^+^* DNA was plotted against the IP/WCE ratio of the 5 h *rad50S* DNA; these are the times of maximal DSB levels in the two strains (Figure 1 [5],[12]). Essentially every probe that showed enrichment (high normalized IP/WCE ratio) in one strain was enriched in the other (Figures 5A and S6A). There is a clear quantitative, positive correlation between the IP/WCE ratios (Figure S9A) of these enriched (DSB hotspot) probes across the two experimental conditions, consistent with the data in Figure 4. Background probes showed no such quantitative correlation. Probes showing enrichment in the *rad50S* 5 h (induced) DNA (*i.e.*, DSB hotspot probes) showed, however, no significant enrichment in the *rad50^+^* 0 h (non-induced) DNA (Figures 5B, S6B, and S9A), as expected since DSB hotspots are not apparent in the 0 h data (Figures 3 and S5). The subset of enriched probes in the 5 h *rad50S* and 3.5 h *rad50^+^* conditions was consistent across the two independent inductions of *rad50S* and *rad50^+^* (Figure S10). These data indicate that there are no obvious regions of the *S. pombe* genome where DSB hotspots occur in *rad50^+^* but not in *rad50S* strains.

Compared to the correlation between the *S. pombe rad50^+^* and *rad50S* meiotic datasets, the correlation between the *RAD50* (*dmc1Δ*) and *rad50S* enrichment ratios of *S. cerevisiae* is much weaker (Figures S6C, S6D, and S9B). Among probes showing enrichment, there are many probes that have a higher, and often much higher, enrichment ratio in *RAD50* (*dmc1Δ*) meiosis than in *rad50S* meiosis, as well as other probes that show similar high enrichment ratios in both. This is expected, given loci where DSBs are frequent in both *RAD50* (*dmc1Δ*) and *rad50S* meiosis and other loci where DSBs are frequent only in *RAD50* (*dmc1Δ*) [8],[9].

As another way of comparing the meiotically induced *rad50S* and *rad50^+^* data from *S. pombe*, we identified regions of significant ChIP enrichment using ChIPOTle [21], with a *p* value cutoff of 0.001. Due to the accumulation of Rec12-DNA intermediates, Rec12 ChIP enrichment over background should be greater in the *rad50S* experiments. As the *p* value that ChIPOTle attaches to peaks is dependent on their degree of enrichment over background, peaks can be detected with greater sensitivity in the *rad50S* experiments. Therefore, for any given significance threshold, if the same pattern of DSBs occurs in both the *rad50S* and *rad50^+^* experiments, we expect some peaks (the stronger ones) to be detected in both sets of experiments but other peaks (the weaker ones) to be detected only in the *rad50S* experiments. This is what we observed. Combining the two independent inductions (Datasets S1 and S2), an average of 10.2% and 5.0% of the genome was enriched (*i.e.*, within ChIPOTle-determined peaks) in the 5 h *rad50S* and the 3.5 h *rad50^+^* data, respectively, but 4.9% of the genome was enriched in both. Therefore, there is no significant class of peaks identified in the *rad50^+^* data that do not have equivalents in the *rad50S* data. In contrast, in *S. cerevisiae* [8] 63% of the genome was enriched in the *RAD50* (*dmc1Δ*) strain, and 32% in the *rad50S* strain, but 31% of the genome was enriched in both. Therefore, in *S. cerevisiae* there is a significant class of probes that are enriched only in the *RAD50* (*dmc1Δ*) background, as well as probes that are enriched in both backgrounds.

A simpler consideration of the ChIPOTle analysis leads to the same conclusion. In our *S. pombe* data, an average of 255 significant peaks was detected in the two 3.5 h *rad50^+^* datasets, and 427 in the two 5 h *rad50S* datasets. Essentially all (94%) of the *rad50^+^* peaks were present in the corresponding *rad50S* datasets (*i.e.*, the peaks overlap), but only 48% of *rad50S* peaks were present in the *rad50^+^* dataset. That is, there are almost no peaks detectable in the *rad50^+^* background that are not detected in the *rad50S* background. The larger number of peaks identified in the *rad50S* background is expected from the greater peak detection sensitivity of ChIPOTle using the *rad50S* dataset, as discussed above. For probes showing enrichment in either the 3.5 h *rad50^+^* or 5 h *rad50S* datasets, the *rad50S* enrichment ratio is consistently ∼3 fold higher than the *rad50^+^* enrichment ratio (Figure S7A). In comparison, the data from *S. cerevisiae* [8] look very different. Here, 95% of 2010 *rad50S* peaks overlap with *RAD50* (*dmc1Δ*) peaks but only 60% of 1816 *RAD50* (*dmc1Δ*) peaks overlap with *rad50S* peaks. That is, there is a substantial number of loci (hotspots) where significant DNA breakage is seen in the *RAD50* (*dmc1Δ*) strain but not in the *rad50S* strain, as well as other loci where significant breakage is seen in both strains (Figure S7B).

## Discussion

Our detection, for the first time, of Rec12-DNA covalent linkages in recombination-proficient (*rad50^+^*) cells allowed us to compare the genome-wide distribution of these linkages, and hence meiotic DSBs, in *rad50^+^* strains and the more thoroughly studied *rad50S* strains. Our results show that the genomic distributions of *S. pombe* meiotic DSBs in these strains are indistinguishable (Figures 1, 3, 4, S2, S5, and [5],[12]). In addition to confirming our previous meiotic DSB map [5], our results have additional implications about the regulation of DSB formation and differences in this regulation among species, as discussed below.

### Different DSB Regulation by the MRN Complex in *S. pombe* and *S. cerevisiae*

An analysis of DSBs by ChIP of the Spo11 protein in a *rad50^+^* meiosis in budding yeast has not been successful [13],[14; M. Lichten, personal communication], presumably because Spo11 is rapidly removed from the DSB 5′ ends. The success of our Rec12-ChIP analysis in fission yeast *rad50^+^* strains (Figures 2, 3, and S5) suggests that the Rec12 protein remains linked to DNA for a longer period of time in fission yeast than does Spo11 in budding yeast. However, even in fission yeast, Rec12 appears to be removed in a rather short period – a DNA sample taken 30 min after the 3.5 h DNA sample studied here (Figures 2, 3, and S5) and similarly analyzed on a microarray showed no discernible difference genome-wide from the 0 h pre-meiotic DNA sample (unpublished data). In addition, multiple assays for Rec12-DNA by PCR at selected loci show that the Rec12-DNA species diminishes substantially between 3.5 and 4 h (Figure 2 and unpublished data). Thus, the first step of DSB repair (Rec12 removal) begins about 30 min after DSB formation (which occurs at about 3 h after meiotic induction) and about 30 min before joint DNA molecules (single Holliday junctions) are first detected [22]. The time between DSB formation and joint molecule detection in *S. cerevisiae* is also about 1 h [e.g., 23]. We infer that in *S. pombe* the Rec12-DNA complex persists until the nuclease for its removal, perhaps the MRN (Mre11-Rad50-Nbs1) complex, binds and acts on this intermediate. In *S. cerevisiae* this step may be very fast.

Why does the *rad50S* mutation behave differently in these two yeasts? The answer may lie in the differential dependence on the MRN (MRX in *S. cerevisiae*) complex for DSB formation in these two distantly related yeasts. *S. cerevisiae rad50Δ* and *mre11Δ* mutants do not form DSBs [24],[25], whereas *S. pombe rad32Δ* (*mre11* homolog) and *rad50Δ* mutants form DSBs with the same kinetics as *rad50S* mutants, although none of these mutants repair the DSBs [26]. The dependence on MRX for DSB formation in budding yeast likely reflects its Spo11-dependent binding at sites of DSBs [13], where it is then also in position to quickly remove the Spo11 protein from the DNA. Since fission yeast lacks this MRN requirement for DSB formation, MRN may be recruited only after DSBs are formed, allowing for a greater life-span of Rec12-DNA complexes. The initial steps of DSB repair – the removal of Rec12 (Spo11) and resection to form invasive ss DNA ends [6] – by MRN and other proteins are thought to be similar in both organisms.

The *rad50S* mutation commonly used in both organisms changes the same amino acid of the protein (Lys^81^→Ile^81^) [12],[18],[24],[27], but this *rad50S* mutant does not form the full number of DSBs in budding yeast [8],[9]. These observations lead us to suggest that in budding yeast, which requires MRX for DSB formation, the *rad50S* (K81I) mutant is incompetent (or less competent) compared to *RAD50* to activate DSB formation at some sites or regions but not at others. Thus, not all hotspots are revealed in *S. cerevisiae rad50S* (K81I) strains [8],[9]. In *dmc1* mutants, a more complete spectrum of hotspots would, in this view, be activated by the wild-type MRX complex, as observed [8],[9]. In contrast, the lack of MRN requirement for DSB formation in *S. pombe* may be the basis for the *rad50S* mutation having no discernible effect on the distribution of DSBs in fission yeast. The decision to make DSBs is made before MRN's meiotic activity on DNA, making MRN unnecessary for the formation – but not the processing – of meiotic DSBs. Thus, in *S. pombe* the entire spectrum of DSBs, with readily detectable Rec12-DNA complexes, is observed. In *S. cerevisiae* and other species in which Rad50 is required for DSB formation, *rad50* mutants with an amino acid substitution other than Rad50 (K81I) [27] and that accumulate DSBs may also allow a full spectrum of DSBs to be observed.

### *S. pombe* DSB Map Is Non-Congruent with the Genetic Map

Crossovers arising from meiotic recombination are much more uniformly distributed across the genomes of both fission yeast and budding yeast than are the sites of DSBs observed in *rad50S* strains [8],[11],[12],[15]. A recent study by Buhler et al. [8] determined that the non-congruence in *S. cerevisiae* is due at least in part to a lower DSB frequency and more restricted DSB distribution in a *rad50S* strain than in a *dmc1Δ* strain, which appears to be more representative of wild-type meiosis. Our results in wild-type (*rad50^+^*) *S. pombe* meiosis reveal the same DSB pattern as that seen in earlier studies of *rad50S* mutants [5],[12]: meiotic DSBs are preferentially located in large intergenic DNA regions and are separated by long distances (∼65 kb on average) where no DSBs are apparent. Studies of wild-type (*rad50^+^*) meiosis have in the past been problematic, primarily because the repair of DSBs in wild-type strains prevents all of the meiotic DSBs from being analyzed and low-level breaks can be missed. While there may be low-level DSBs dispersed across the *S. pombe* genome and not detected in our analysis, it is clear that there are essentially no DSB hotspots in *rad50^+^* that are not present in *rad50S* (Figures 3, 5, and S5).

In *S. pombe*, some intervals with no detectable DSBs nevertheless contain abundant crossovers [5],[12],[15]. The 0.5 Mb region of *Not*I fragment J on chromosome I has been extensively studied both genetically for crossovers and physically for DSBs [12]. The number of DSBs detected in this interval – about one DSB per four DNA molecules in a meiotic cell – is not enough to account for the crossovers that occur on this fragment – about one per meiotic cell – since there are about three times more intersister (genetically silent) exchanges than interhomolog exchanges, at least at the major DSB hotspot *mbs1* on that fragment [15]. In the 57 kb *res2* – *ura1* subregion of *Not*I fragment J there are ∼0.08 crossovers, over 10 times more than predicted by the <0.005 DSBs per meiotic tetrad [12; unpublished data]. It had been suggested that crossovers in such regions might arise from ss nicks [15], but since all meiotic crossovers are dependent on Rec12 [28], we would expect even sites of nicks to have Rec12 covalently linked to the DNA and therefore enriched by ChIP. Ludin et al. [29] analyzed by microarrays the genome-wide distribution of Rec12 after it was formaldehyde-crosslinked to DNA and found a more uniform distribution than we find for Rec12 self-linked to DNA. Although much of the Rec12 detected with formaldehyde-crosslinking does not make detectable DSBs, this population of Rec12 may nevertheless be required for crossovers in DSB-poor regions. Although the basis of the DSB–crossover discrepancy remains undetermined, our results rule out one explanation – that DSBs are underrepresented in *rad50S* strains.

### Suitability of *rad50S* Strains for DSB Analysis in Other Species

Results from the DSB analysis of a *dmc1Δ* mutant in *S. cerevisiae* [8],[9] have brought into question the reliability of DSB maps generated using the *rad50S* mutation. Our results in *S. pombe* question whether these findings from budding yeast apply to other organisms. *rad50S*-like mutations may reveal the wild-type distribution in other species, particularly those in which Rad50 is not required for DSB formation, such as *Arabidopsis thaliana*, *Drosophila melanogaster*, *Coprinus cinereus*, and perhaps *Caenorhabditis elegans* [30],[31],[32; M. Zolan, personal communication]. In species that appear not to have a Dmc1 ortholog a microarray analysis of DSBs performed with a *rad50S*-like mutant may be the most feasible method to reveal the DSB distribution. Our results indicate that in these cases the results may reflect those in wild type. Regardless of the genetic background used and methodology chosen, understanding where meiotic DSBs occur and what DNA characteristics influence DSB location remains an important question in understanding the regulation of meiotic recombination.

## Materials and Methods

### *S. pombe* Strains

Strains used were GP1979 (*h^−^*/h^−^ ade6-52/ade6-M26 lys3-37/+ +/ura1-171 pro1-1/+ pat1-114/pat1-114 end1-458/end1-458), *GP3718* (h^+^ ade6-3049 pat1-114 rad50S end1-458), *GP6203* (h^−^/h^−^ ade6-3049/ade6-3049 pat1-114/pat1-114 rad50S/rad50S rec12-201::6His-2FLAG/rec12-201::6His-2FLAG +/his4-239 lys4-95/+), and *GP6232* (h^−^/h^−^ ade6-3049/ade6-3049 pat1-114/pat1-114 rec12-201::6His-2FLAG/rec12-201::6His-2FLAG +/his4-239 lys4-95/+). Alleles were described previously [5],[18],[19],[26].

### Meiotic DNA Preparation and Southern Blot Analysis

To assess events in *S. pombe* meiosis, we used strains carrying the temperature-sensitive *pat1-114* mutation, which affords high synchrony but has no detectable effect on DSB formation or location [5],[33]. Cultures were grown to mid log-phase and starved for nitrogen to arrest cells in the G1 phase of the cell cycle; nitrogen was restored and the temperature raised to initiate meiosis. Cells were harvested, embedded in agarose plugs, and treated with enzymes to lyse the cells and to partially purify the DNA. After digestion with *Not*I restriction enzyme, the DNA was subjected to pulsed-field gel electrophoresis and Southern blot hybridization. These methods are detailed elsewhere [12],[34]. The probe used on *Not*I fragment K (Figure 1A and S2A) extends from bp 3600336 to bp 3601359 on chromosome I; the probe used on *Not*I fragment D (Figure 1B and S2B) extends from bp 1025344 to bp 1026300 on chromosome I (accession # NC_003424.3).

### Preparation of Meiotic Chromatin, Rec12 ChIP, and Microarray Analysis

Strains with both *rad50^+^* and *rad50S* genetic backgrounds were induced twice. Chromatin was prepared, immunoprecipitated, assayed by locus-specific PCR, and analyzed on microarrays as described [5], except that Agilent Whole Genome 4×44 K *S. pombe* oligonucleotide microarrays were used. The 0 h and 3.5 h *rad50^+^* DNA and the 5 h *rad50S* DNA were analyzed on microarrays twice; the 4 h *rad50^+^* DNA was analyzed only once, as was the 0 h *rad50S* DNA, which confirmed earlier results [5].

### Identification of Rec12 Enrichment peaks

Regions of significant enrichment were identified using the Gaussian setting of ChIPOTle (v 1.0) [21] with a *p* value cutoff of 0.001.

## Supporting Information

Figure S1 FACS Analysis Shows the Majority of Cells Were in G1 Phase after Nitrogen Starvation at the Start of Meiosis in Both Strains GP6203 and GP6232. After meiotic induction by the addition of nitrogen and shift to high temperature, each strain underwent a nearly synchronous meiosis, with DNA replication occurring between 2 and 3 h.
(0.05 MB PDF)

Figure S2 Southern Blots Reveal Indistinguishable DSB Hotspots in *rad50^+^* and *rad50S* Strains. DNA from meiotically induced strains was digested with *Not*I, and the fragments separated by pulsed-field gel electrophoresis. Inductions were performed concurrently with *rad50^+^* (GP1979; left) and *rad50S* (GP3718; right) strains. (A) The blots were probed on the left end of the 480 kb *Not*I restriction fragment K of chromosome I. (B) The same blots were probed on the left end of the 1.2 Mb *Not*I restriction fragment D of chromosome I. On the right are lane traces of the time of maximal DSBs [3.5 h *rad50^+^* (dark lines) and 5 h *rad50S* (light lines)] for each probing. Data from *rad50^+^* are from an induction independent of that shown in Figure 1. Independent data for *Not*I fragment D from *rad50S* are shown in Cromie et al. [5].
(4.6 MB TIF)

Figure S3 Rec12 Is Bound to DSB Ends in Both *rad50^+^* and *rad50* Strains. DNA from meiotically induced *rad50^+^* (GP6232) and *rad50S* (GP6203) cells was extracted either with or without Proteinase K digestion followed by phenol-chloroform extraction, ethanol precipitation, *Mlu*I digestion, and ethanol precipitation, similar to the method of Keeney et al. [20]. The DNA was separated by pulsed-field gel electrophoresis and analyzed by Southern blot hybridization. Substantially more meiotically broken (DSB) DNA was recovered from both the *rad50^+^* (left panels) and *rad50S* (right panels) strains with Proteinase K digestion than without, indicating that a significant amount of DSB DNA was bound by Rec12 in both cases. Quantitation of the gels is shown on the far right. The *Mlu*I restriction fragments with *ade6-3049* (top panels) and *mbs1* (bottom panels) are 28.2 and 20.9 kb, respectively. The probe for *ade6-3049* extends from bp 1309506 to bp 1310549 on chromosome III (accession # NC_003421.2); the probe for *mbs1* extends from bp 768436 to bp 769496 on chromosome I (accession # NC_003424.3).
(0.3 MB PDF)

Figure S4 Rec12 IP/WCE Enrichment Is Seen Only in the 5 *rad50S* h and 3.5 h *rad50^+^* Data. Quantile-quantile (Q-Q) plots are of IP/WCE hybridization ratios (log_10_) for Dataset S2 versus simulated normal values (with a mean of 0 and variance of 1). Normally distributed log IP/WCE hybridization ratios should result in a straight line passing through the origin. Note that the 0 h *rad50^+^* data closely follow this background expectation, while the 5 *rad50S* h and 3.5 h *rad50^+^* data have many high IP/WCE ratios clearly above those expected from the normal distribution, as expected for Rec12-DNA enrichment at linkage sites.
(0.1 MB PDF)

Figure S5 Rec12-DNA Linkages across the Entire *S. pombe* Genome. Shown are the median-normalized IP/WCE hybridization ratios from experiment 2 (Dataset S2). Data from induced cells (*rad50^+^* strain GP6232 at 3.5 h after meiotic induction and *rad50S* strain GP6203 at 5 h) are in red. Data from uninduced (0 h) cells (*rad50^+^* strain GP6232) are in blue. Where peaks go off-scale, the peak maximum is indicated. The data are neither smoothed nor filtered for spurious values, except for removal of 25 data points for ∼10.7 kb of DNA deleted in the *rad50S* strain GP6203 between direct repeats at bp 2929282–2931720 and 2939711–2942292 on chromosome I (Accession: NC_003424.3) (unpublished data). These ∼2.5 kb repeats have identities at both ends but an ∼150 bp internal region of non-homology. These 25 data points have spuriously low hybridization values for DNA from the WCE, as expected for a deletion. The strong peak seen in the 0 h data for chromosome III occurs at the site of the *ade6-3049* break hotspot. It is not clear why this peak is present in the 0 hr data. It is absent from the 0 hr experiments in Dataset S1.
(1.9 MB PDF)

Figure S6 All DSB Hotspots Detected in *rad50^+^* Are Also DSB Hotspots in *rad50S*; In *S. cerevisiae* Microarray Experiments Many Probes Show Greater Meiotic DSB Hotspot Activity in *dmc1Δ* Mutants Than in *rad50S* Mutants. The IP/WCE ratio of each probe in the *rad50^+^* microarray hybridization is plotted against the IP/WCE ratio of the same probe in the 5 h *rad50S* microarray hybridization. The plots are on a log scale. Color indicates density of plotted points with yellow highest and dark blue lowest, calculated by superimposing a grid with spacing 10 ^0.01^ on the chart (log_10_ enrichment values) and coloring all points within each grid square based on the number of points in that square. (A) The 3.5 h *rad50^+^* IP/WCR ratios are positively correlated with those of the 5 h *rad50S* data. All probes enriched by IP of the *rad50^+^* DNA are enriched in the *rad50S* DNA and *vice versa*. (B) The uninduced (0 h) *rad50^+^* IP/WCR ratios show no correlation with those of *rad50S*, as expected for uninduced background signals. These data are from experiment 1 (Dataset S1); similar results were obtained in an independent induction of each strain (Figure 5). The number of points per grid square ranged from 1 to 37 in A and 1 to 19 in B. For comparison with *S. cerevisiae*, we replotted data from [8]. The normalized DSB enrichment ratio of each probe in the meiotic *dmc1Δ* microarray hybridization (C) or meiotic *dmc1Δ spo11-Y135F* (inactive Spo11) microarray hybridization (D) is plotted against the enrichment ratio of the same probe in the meiotic *rad50S* microarray hybridization. The plots are on a log scale. Some probes show similar enrichment in the *dmc1Δ* and *rad50S* datasets, while others are much more highly enriched in the *dmcΔ* dataset (C). No probes enriched in the *rad50S* dataset show significant enrichment in the negative control *dmc1Δ spo11-Y135F* dataset (D). Color indicates density of plotted points as above. The number of points per grid square ranged from 1 to 22 in C and 1 to 6 in D.
(2.7 MB PDF)

Figure S7 Enriched Probes Are Consistently ∼3-Fold More Highly Enriched in the 5 h *rad50S* Datasets of *S. pombe* Compared to the 3.5 h *rad50^+^* Datasets; In Contrast, Many Probes Show Enrichment Only in the Meiotic *dmc1Δ* Datasets and Not the *rad50S* Datasets of *S. cerevisiae*. (A) A frequency histogram of the log_10_ [(3.5 h *rad50^+^* value)/(5 h *rad50S* value)] for probes showing enrichment (Rec12 IP/WCE≥10) in either the *S. pombe* 5 h *rad50S* (top) or the 3.5 h *rad50^+^* (bottom) conditions from Dataset S2 is shown. In both cases, the IP/WCE ratios are consistently ∼3-fold higher in the *rad50S* condition (average log_10_ ratio between conditions of ∼−0.5). A similar result was obtained using Dataset S1 (data not shown). (B) A frequency histogram of the log_10_ [(*dmc1Δ* value)/(*rad50S* value)] for probes showing enrichment (enrichment ratio≥10) in either the *rad50S* (top) or the *dmc1Δ* (bottom) conditions from [8] is shown. For probes showing enrichment in the *rad50S* condition, enrichment ratios are similar in the *rad50S* and *dmc1Δ* conditions (average log_10_ ratio between conditions of ∼0). In contrast, for many probes showing enrichment in the *dmc1Δ* condition, the *rad50S* enrichment ratio is much lower, giving significantly higher average log ratios between conditions.
(0.1 MB PDF)

Figure S8 Full-length DNA Strands with 5′ and 3′ Ends at DSBs Appear in *rad50^+^* Meiosis. DNA from meiotically induced cells with the *ade6-3049* hotspot was treated with Proteinase K, digested with *Afl*II, electrophoresed through an alkaline agarose gel (50 mM NaOH, 1 mM EDTA), and analyzed by Southern blot hybridization using two probes for the right end of the 6.6 kb *Afl*II fragment containing *ade6*. Each probe was specific for either the strand with 3′-ends or the strand with 5′-ends at the DSBs as indicated. DNA from three independent experiments using strains GP6232 (*rad50^+^*) and GP3718 (*rad50S*) harvested at the indicated time after meiotic induction was analyzed in panels A, B, and C. [^32^P]-labelled 1 kb Plus DNA markers (Invitrogen) were run on the gels; white arrowheads indicate the 1 kb fragment. Line traces from phosphorimage analysis are from the times and strains indicated with an asterisk. Note that the peaks with 3′-strand probes (red lines) and 5′-strand probes (blue lines) nearly coincide, indicating that the complementary strands have indistinguishable end points at the DSBs. In DNA from *rad50^+^* DNA the peaks are sharper and more intense with 3′-strand probes than with 5′-strand probes, indicating that the 3′-ends undergo less resection than 5′-ends, as expected from ongoing DSB repair in *rad50^+^* strains.
(0.4 MB PDF)

Figure S9 IP/WCE Ratios for Rec12-Enriched Probes Are Highly Correlated between the 3.5 h *rad50^+^* and 5 h *rad50S* Data; A Much Weaker Correlation Is Seen between the Meiotic DSB Enrichment Ratios of *dmc1Δ* and *rad50S* Mutants of *S. cerevisiae*. (A) Probes were ranked by the 5 h *rad50S* IP/WCE ratio from *S. pombe* Dataset S2 and divided into 20 groups of equal numbers of probes. For each group of probes the Pearson product-moment correlation coefficient (R) was calculated first between the log 5 h *rad50S* and log 3.5 h *rad50^+^* ratio values and then between the log 5 h *rad50S* and log 0 h *rad50^+^* ratio values. R^2^ values, representing the association between the paired ratios, are plotted against the ordered five-centile groupings (where 100 is the 5 h *rad50S* centile with the highest IP/WCE values). (B) Probes were ranked by the meiotic *rad50S* IP/WCE ratio from *S. cerevisiae*, taken from [8], and divided into 20 groups of equal numbers of probes. For each group of probes the Pearson product-moment correlation coefficient (R) was calculated first between the log meiotic *rad50S* and log meiotic *dmc1Δ* ratio values and then between the log meiotic *rad50S* and log meiotic *dmc1Δ spo11-Y135F* (negative control) values. R^2^ values, representing the association between the paired ratios, are plotted against the ordered five-centile groupings (where 100 is the meiotic *rad50S* centile with the highest IP/WCE values).
(0.3 MB PDF)

Figure S10 A Consistent Subset of Probes Shows Rec12 IP/WCE Enrichment across Hybridizations in Both *rad50^+^* and *rad50S* Backgrounds. (A) The 5 h Rec12 IP/WCE ratio of each probe in the second *rad50S* microarray hybridization (Dataset S2) is plotted against the 5 h IP/WCE ratio of the same probe in the first *rad50S* microarray hybridization (Dataset S1). (B) The 3.5 h Rec12 IP/WCE ratio of each probe in the second *rad50^+^* microarray hybridization (Dataset S2) is plotted against the 3.5 h IP/WCE ratio of the same probe in the first *rad50^+^* microarray hybridization (Dataset S1). In both (A) and (B), essentially all probes showing enrichment in one hybridization also show enrichment in the other. The plots are on a log scale.
(0.1 MB PDF)

Dataset S1 Median-normalized Genome-wide IP/WCE Hybridization Ratios from Experiment 1. Data are from strain GP6203 (*rad50S*) before (0 h) and 3.5 h after meiotic induction and from strain GP6232 (*rad50^+^*) before (0 h) and 5 h after meiotic induction.
(7.9 MB XLS)

Dataset S2 Median-normalized Genome-wide IP/WCE Hybridization Ratios from Experiment 2. Data are from strain GP6203 (*rad50S*) before (0 h) and 3.5 h and 4 h after meiotic induction and from strain GP6232 (*rad50^+^*) 5 h after meiotic induction.
(7.8 MB XLS)
