# Supplementary figures and images for: Indistinguishable Landscapes of Meiotic DNA Breaks in rad50+ and rad50S Strains of Fission Yeast Revealed by a Novel rad50+ Recombination Intermediate
Source: PLoS Genet. 2008 Nov 21;4(11):e1000267. doi: 10.1371/journal.pgen.1000267 (PMC2580034; doi:10.1371/journal.pgen.1000267)

**GP 6232 (rad50<sup>+</sup>)**

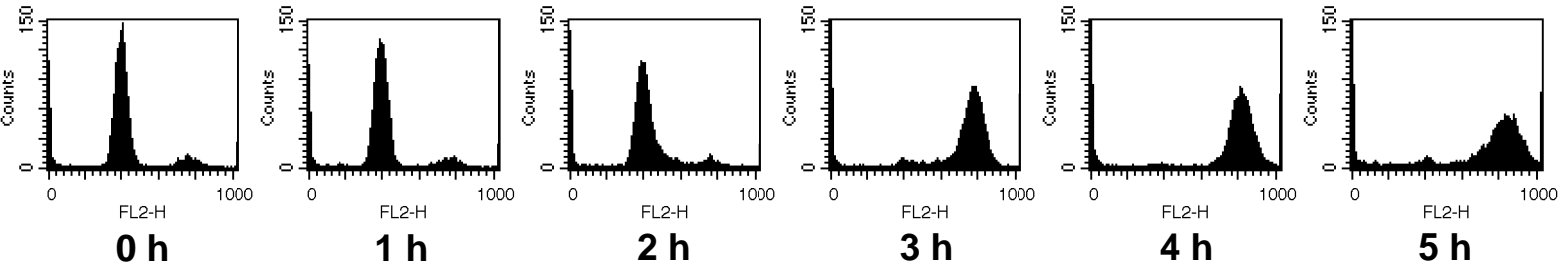

**GP 6203 (rad50S)**

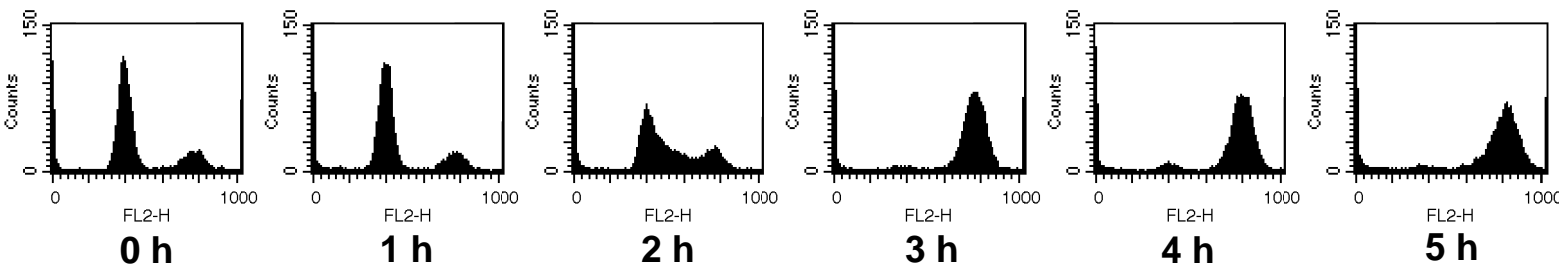

Supplement: Figure S1 — FACS Analysis Shows the Majority of Cells Were in G1 Phase after Nitrogen Starvation at the Start of Meiosis in Both Strains GP6203 and GP6232. After meiotic induction by the addition of nitrogen and shift to high temperature, each strain underwent a nearly synchronous meiosis, with DNA replication occurring between 2 and 3 h. (0.05 MB PDF) [file pgen.1000267.s001.pdf]

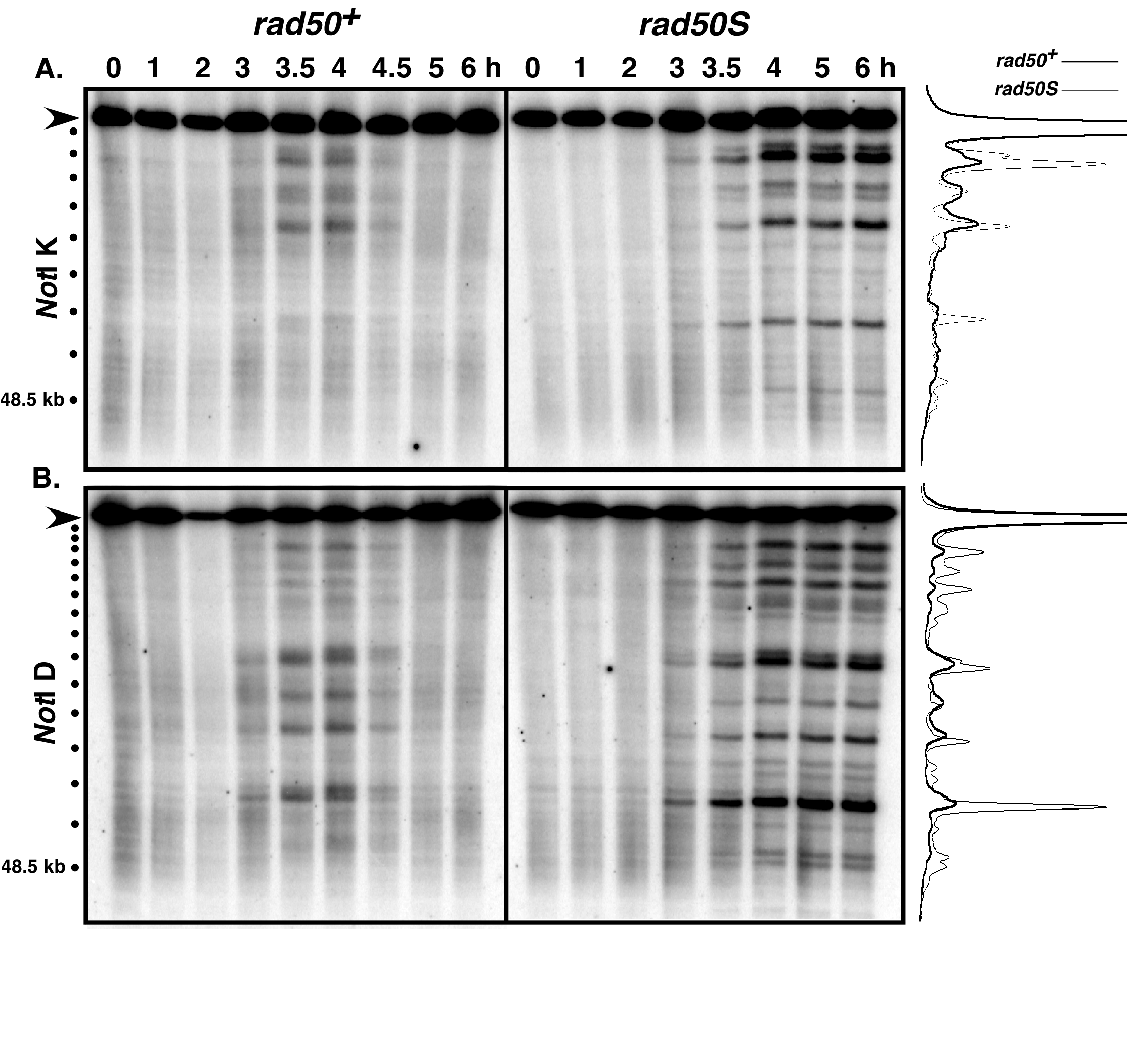

Supplement: Figure S2 — Southern Blots Reveal Indistinguishable DSB Hotspots in rad50+ and rad50S Strains. DNA from meiotically induced strains was digested with NotI, and the fragments separated by pulsed-field gel electrophoresis. Inductions were performed concurrently with rad50+ (GP1979; left) and rad50S (GP3718; right) strains. (A) The blots were probed on the left end of the 480 kb NotI restriction fragment K of chromosome I. (B) The same blots were probed on the left end of the 1.2 Mb NotI restriction fragment D of chromosome I. On the right are lane traces of the time of maximal DSBs [3.5 h rad50+ (dark lines) and 5 h rad50S (light lines)] for each probing. Data from rad50+ are from an induction independent of that shown in Figure 1. Independent data for NotI fragment D from rad50S are shown in Cromie et al. [5]. (4.6 MB TIF) [file pgen.1000267.s002.tif]

*ade6-3049*

*rad50*<sup>+</sup>

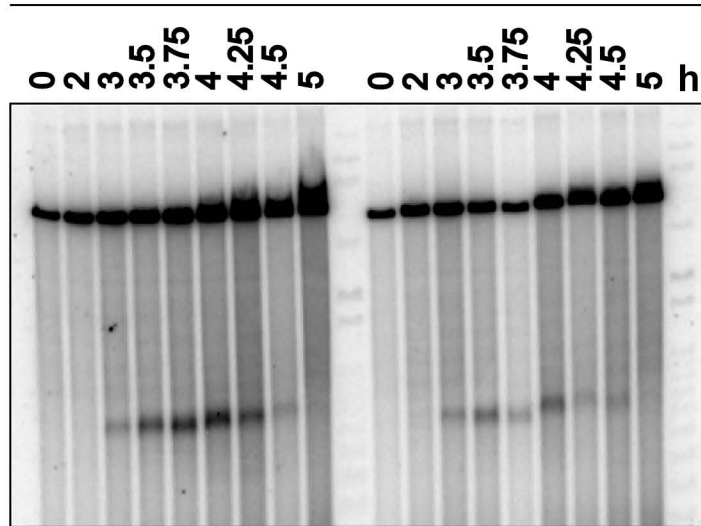

*rad50S*

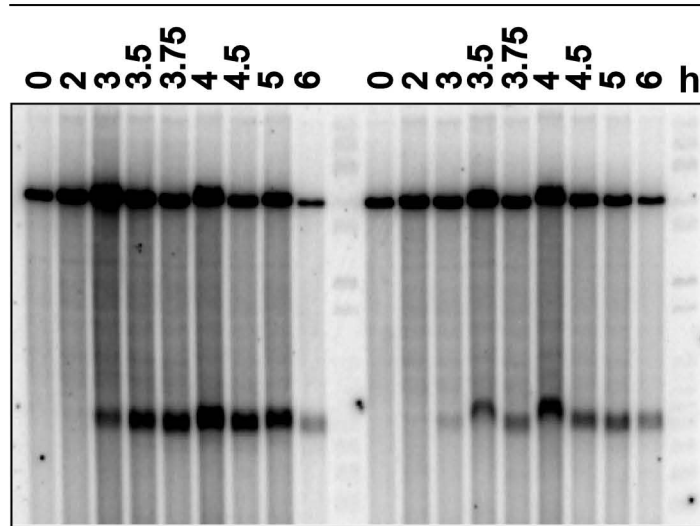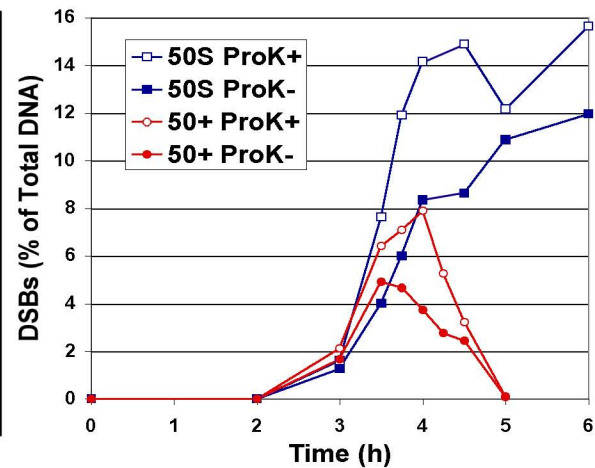

*mbs1*

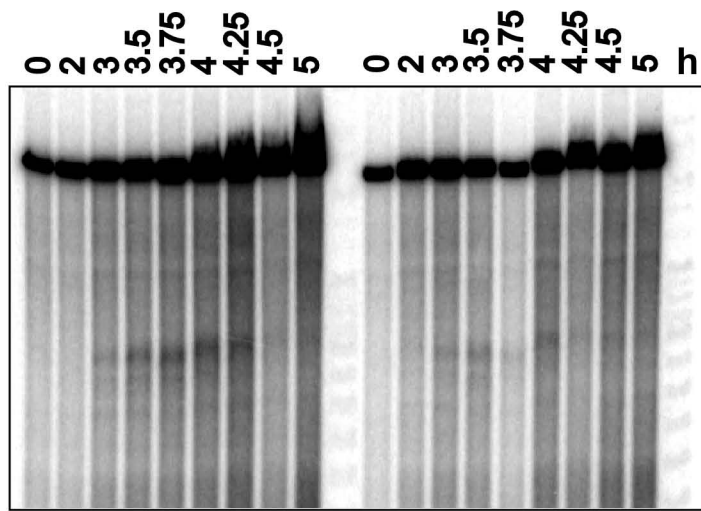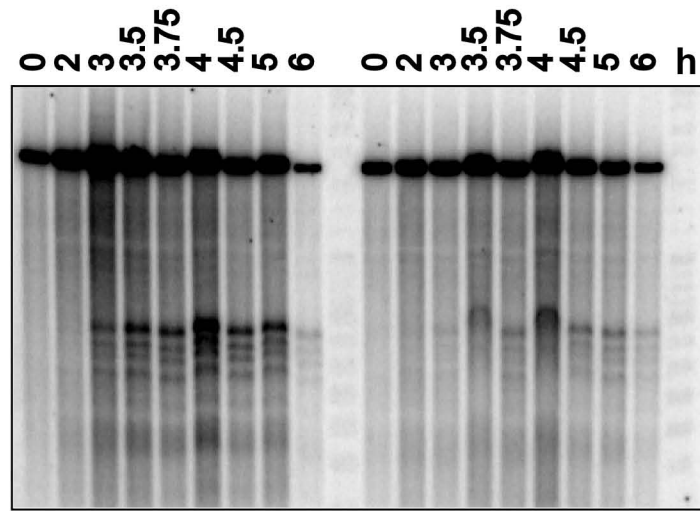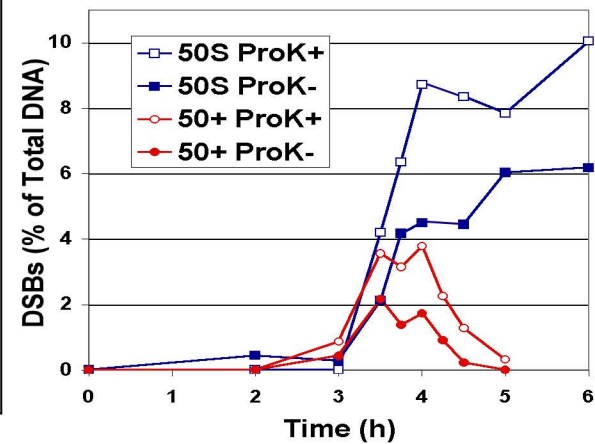

Supplement: Figure S3 — Rec12 Is Bound to DSB Ends in Both rad50+ and rad50 Strains. DNA from meiotically induced rad50+ (GP6232) and rad50S (GP6203) cells was extracted either with or without Proteinase K digestion followed by phenol-chloroform extraction, ethanol precipitation, MluI digestion, and ethanol precipitation, similar to the method of Keeney et al. [20]. The DNA was separated by pulsed-field gel electrophoresis and analyzed by Southern blot hybridization. Substantially more meiotically broken (DSB) DNA was recovered from both the rad50+ (left panels) and rad50S (right panels) strains with Proteinase K digestion than without, indicating that a significant amount of DSB DNA was bound by Rec12 in both cases. Quantitation of the gels is shown on the far right. The MluI restriction fragments with ade6-3049 (top panels) and mbs1 (bottom panels) are 28.2 and 20.9 kb, respectively. The probe for ade6-3049 extends from bp 1309506 to bp 1310549 on chromosome III (accession # NC_003421.2); the probe for mbs1 extends from bp 768436 to bp 769496 on chromosome I (accession # NC_003424.3). (0.3 MB PDF) [file pgen.1000267.s003.pdf]

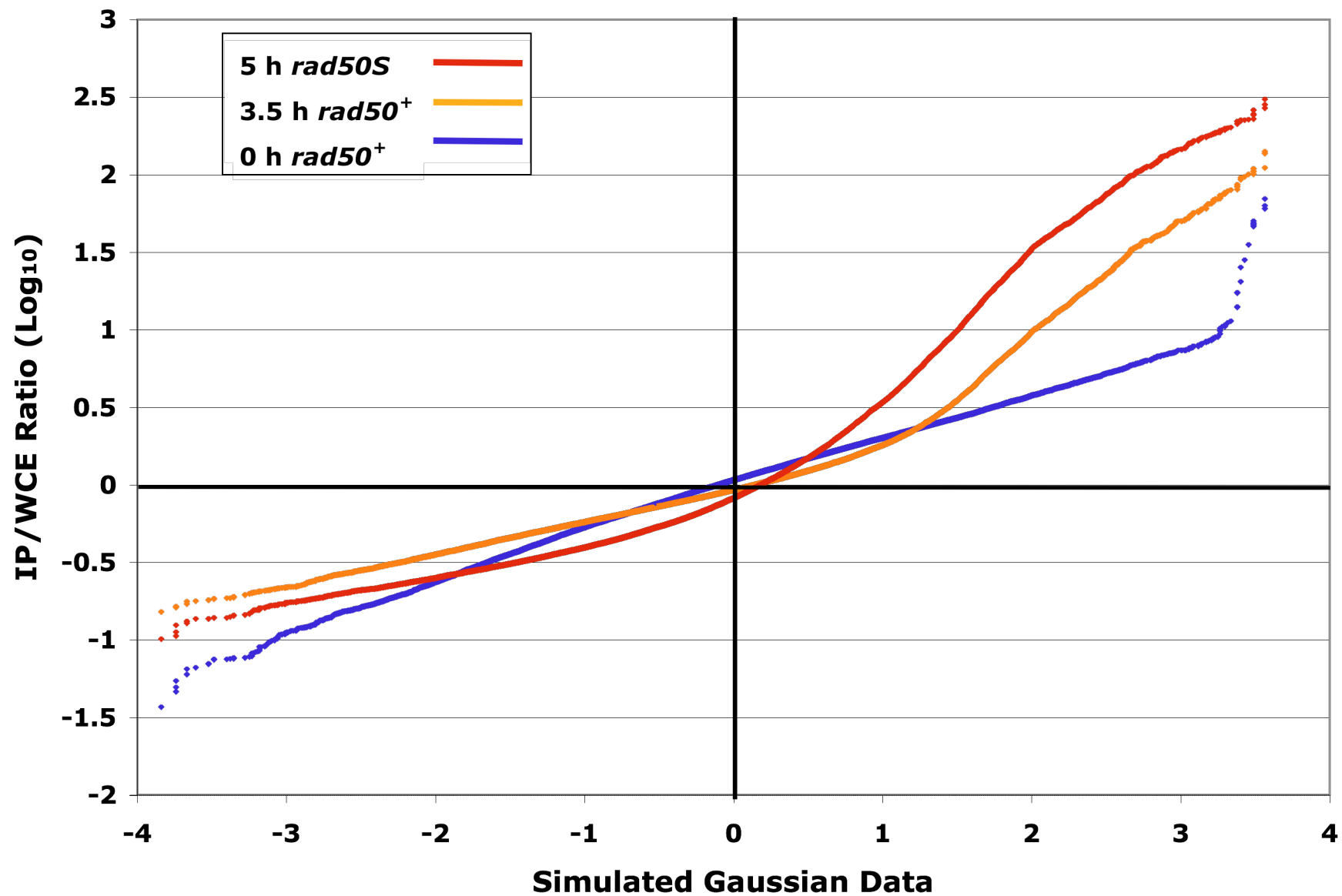

Supplement: Figure S4 — Rec12 IP/WCE Enrichment Is Seen Only in the 5 rad50S h and 3.5 h rad50+ Data. Quantile-quantile (Q-Q) plots are of IP/WCE hybridization ratios (log10) for Dataset S2 versus simulated normal values (with a mean of 0 and variance of 1). Normally distributed log IP/WCE hybridization ratios should result in a straight line passing through the origin. Note that the 0 h rad50+ data closely follow this background expectation, while the 5 rad50S h and 3.5 h rad50+ data have many high IP/WCE ratios clearly above those expected from the normal distribution, as expected for Rec12-DNA enrichment at linkage sites. (0.1 MB PDF) [file pgen.1000267.s004.pdf]

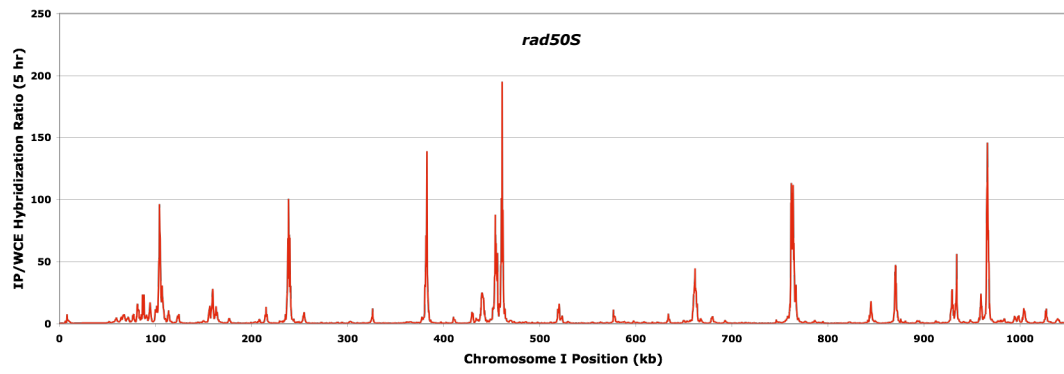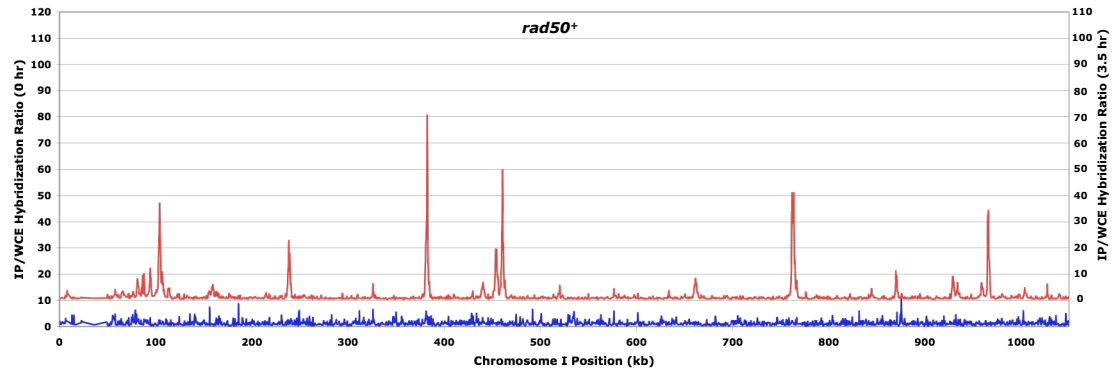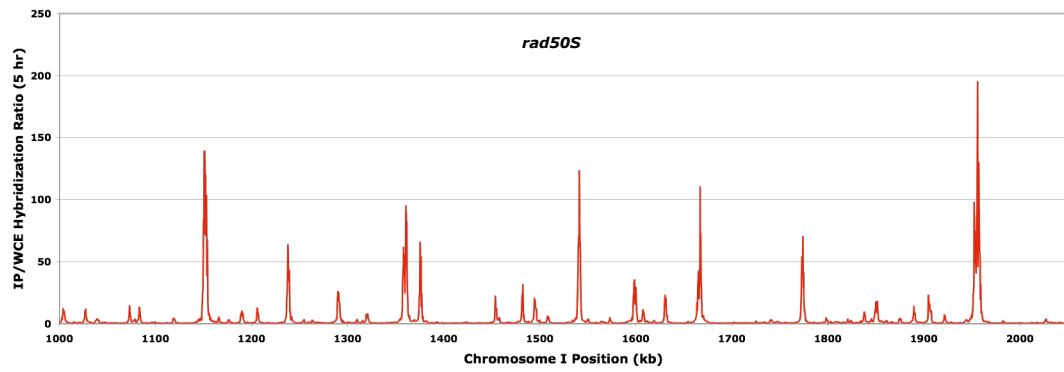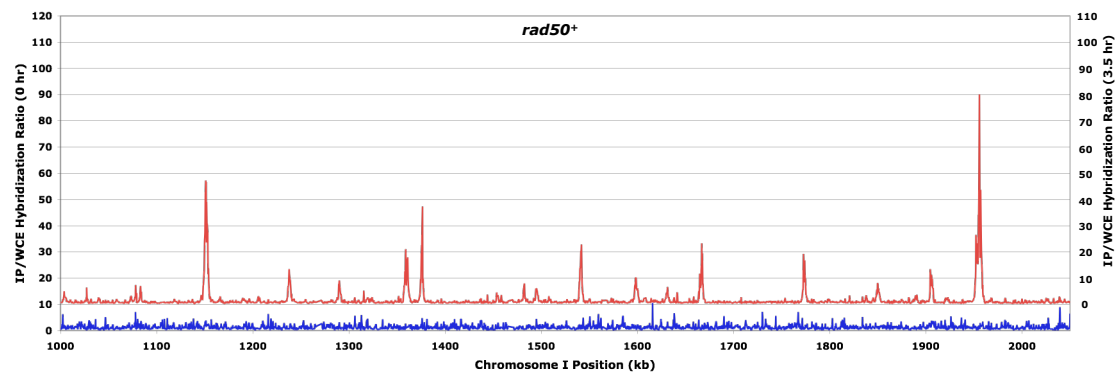

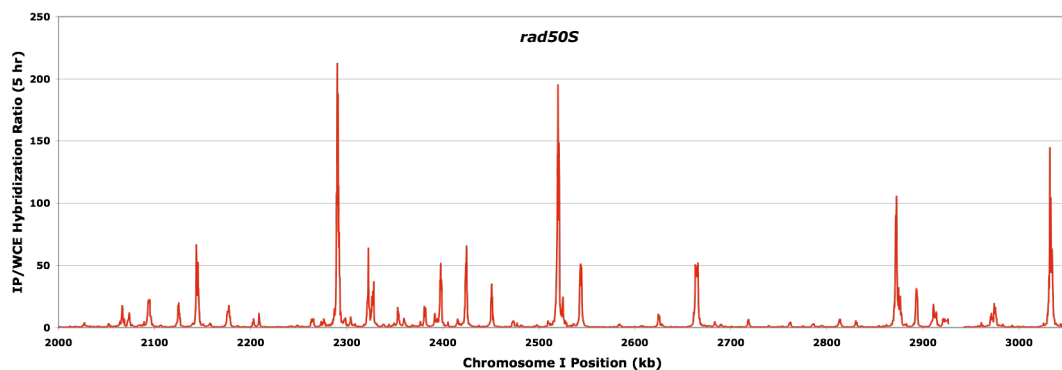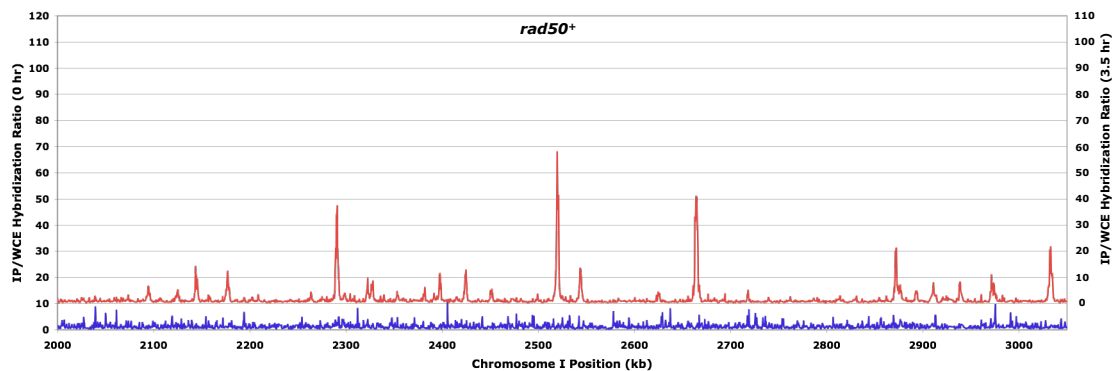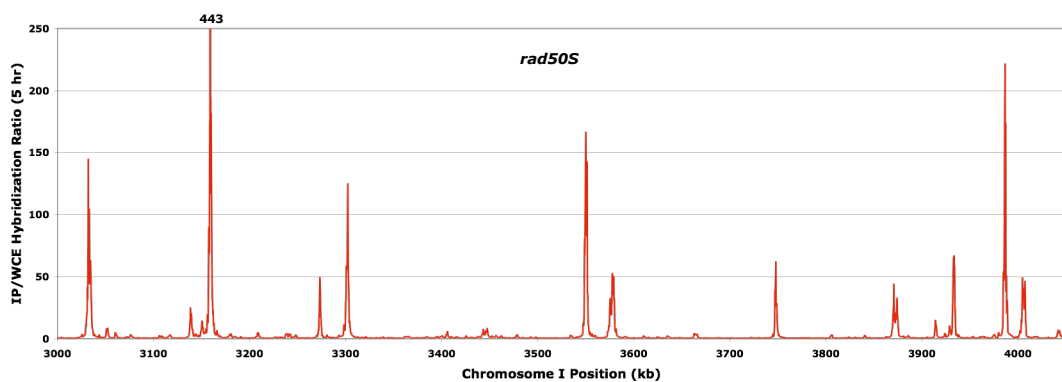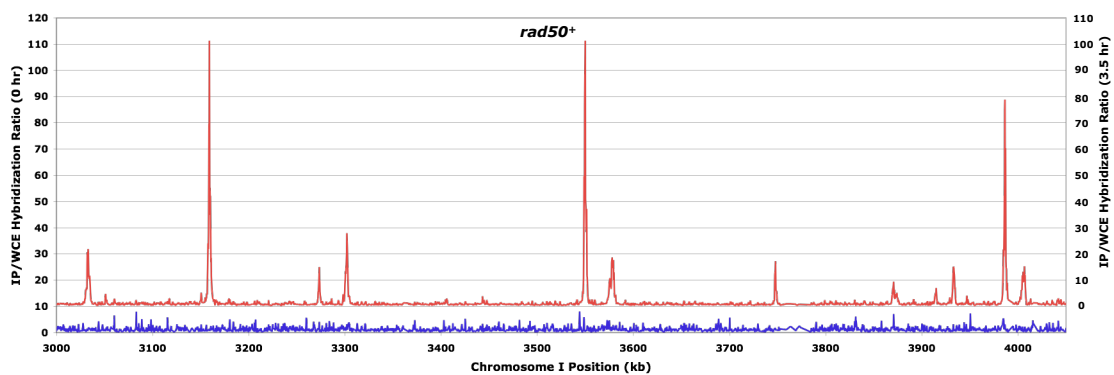

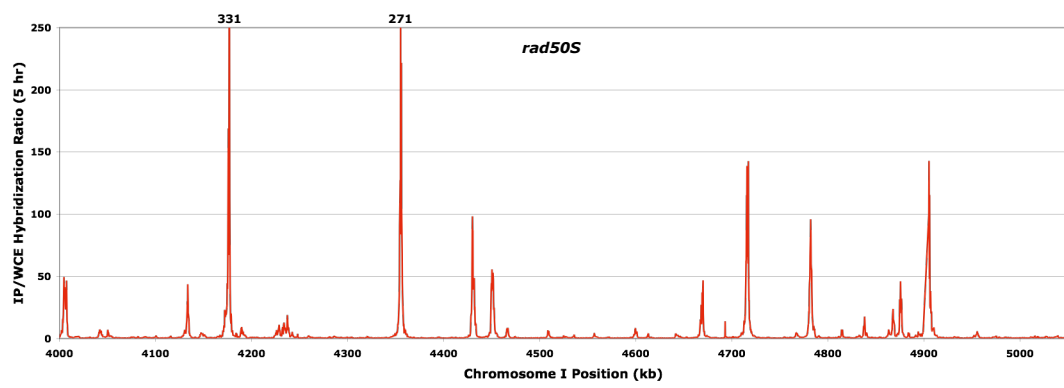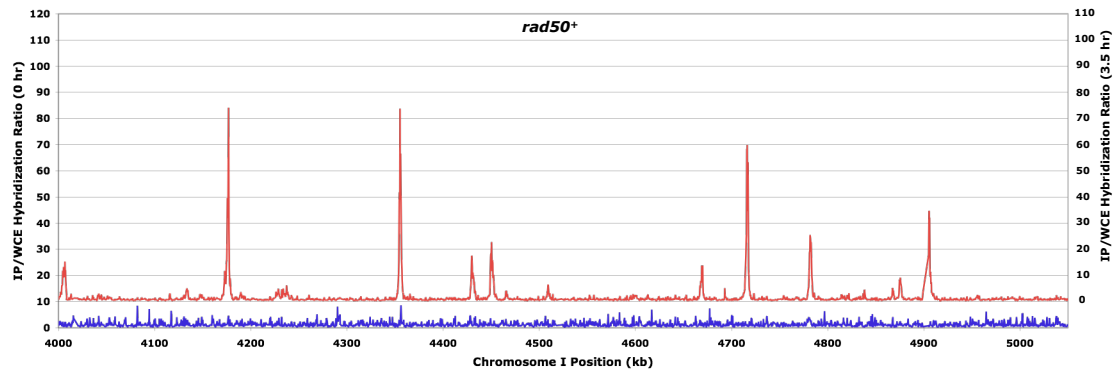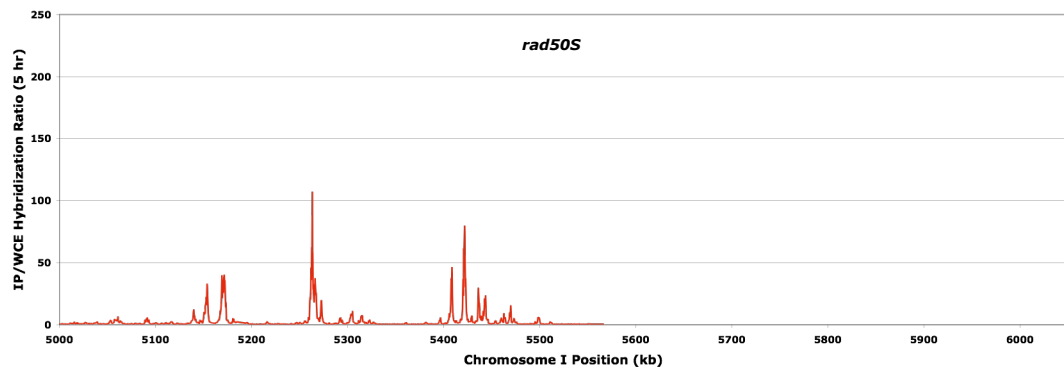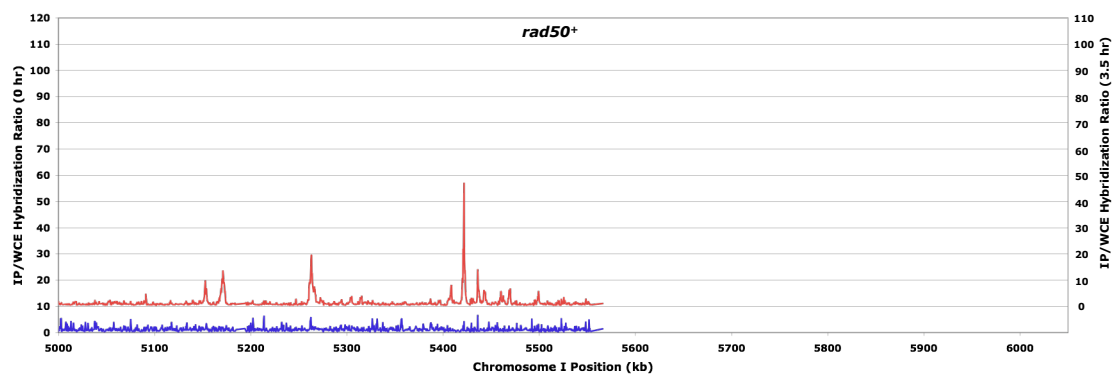

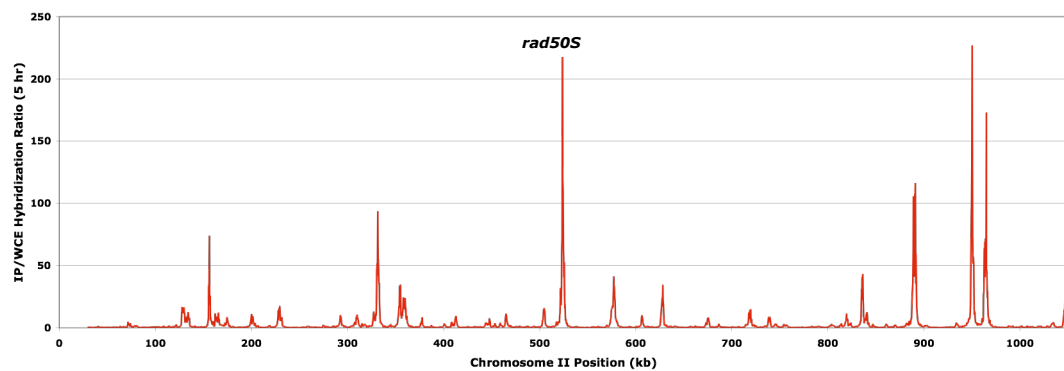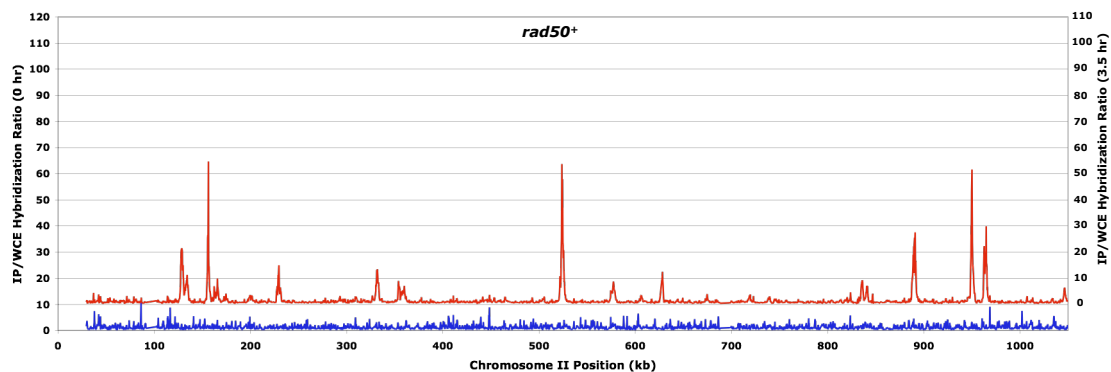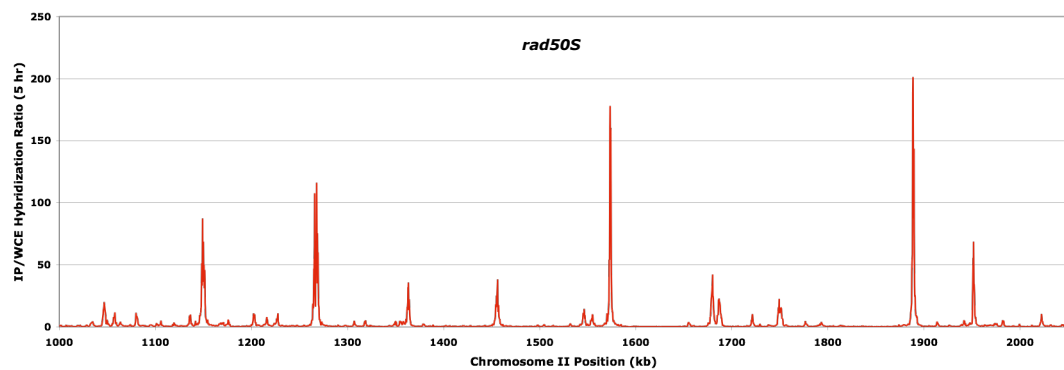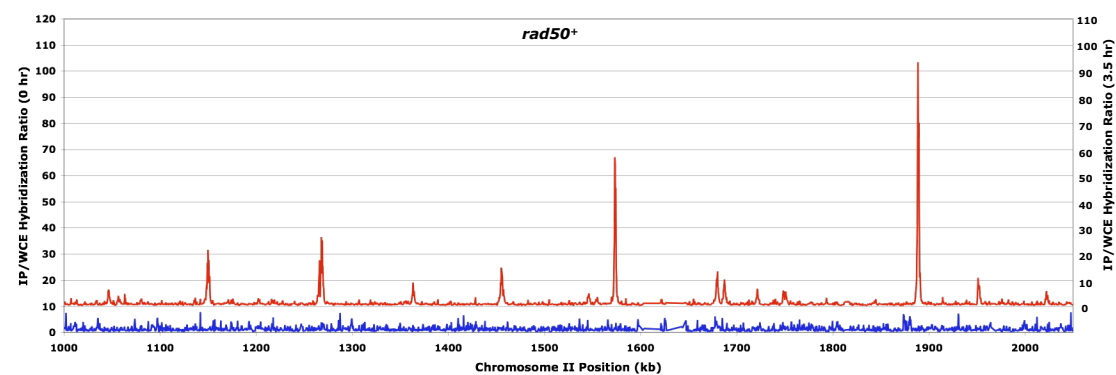

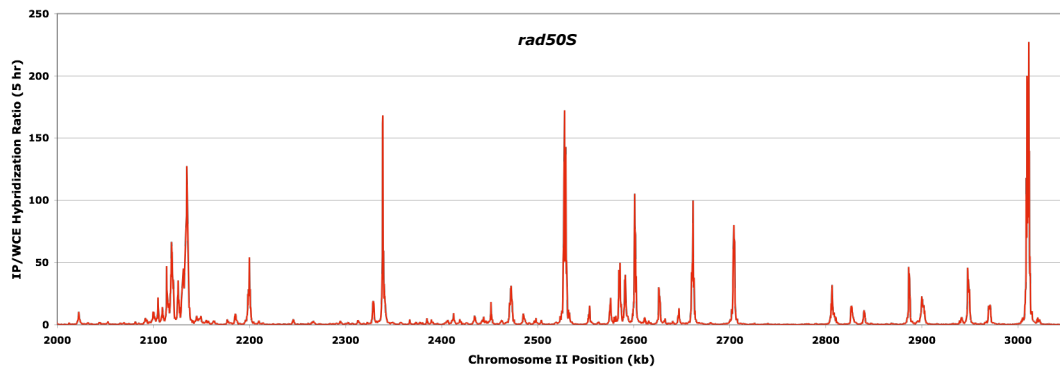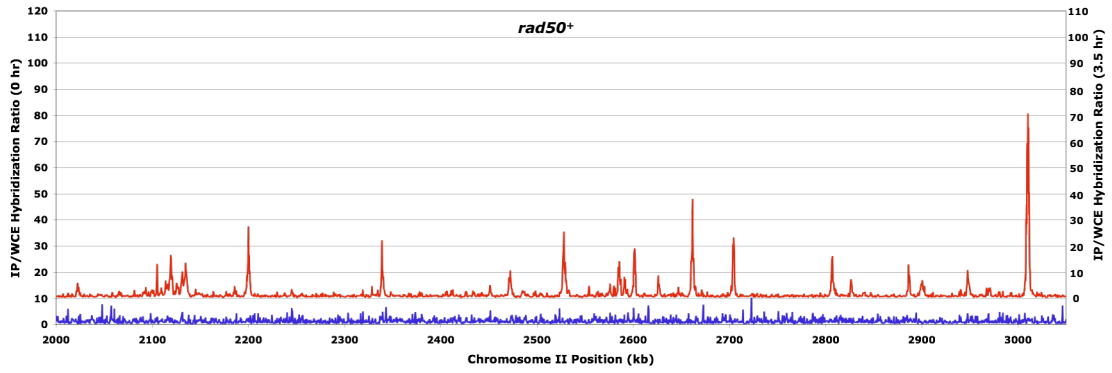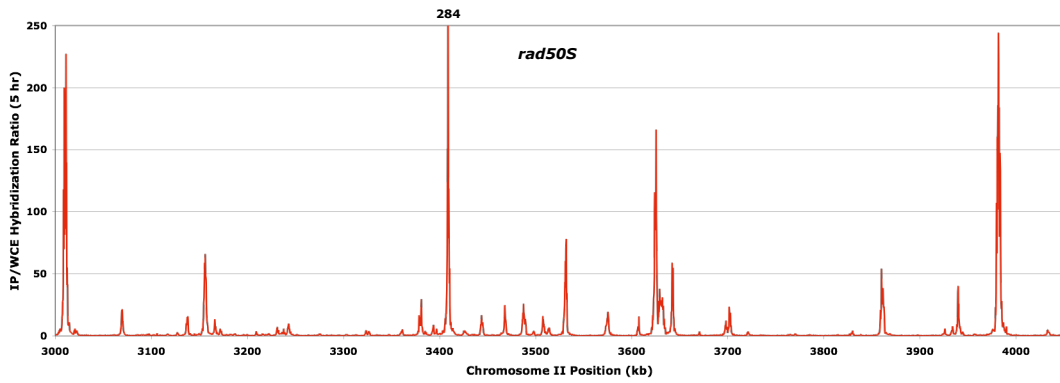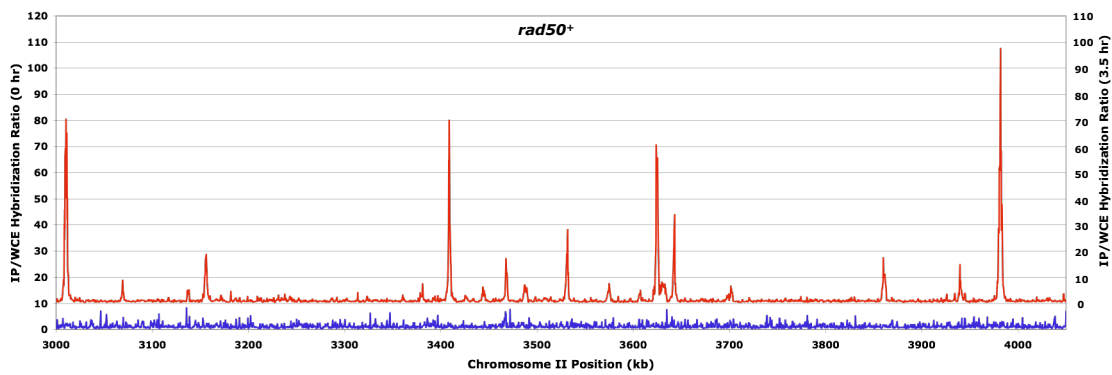

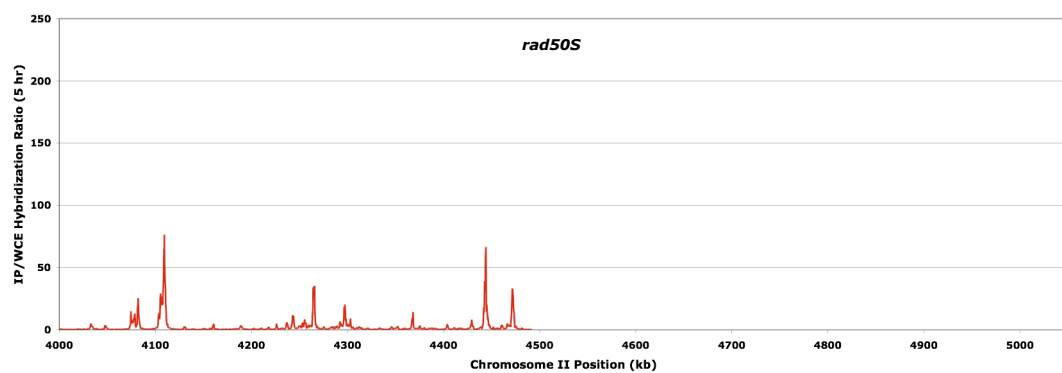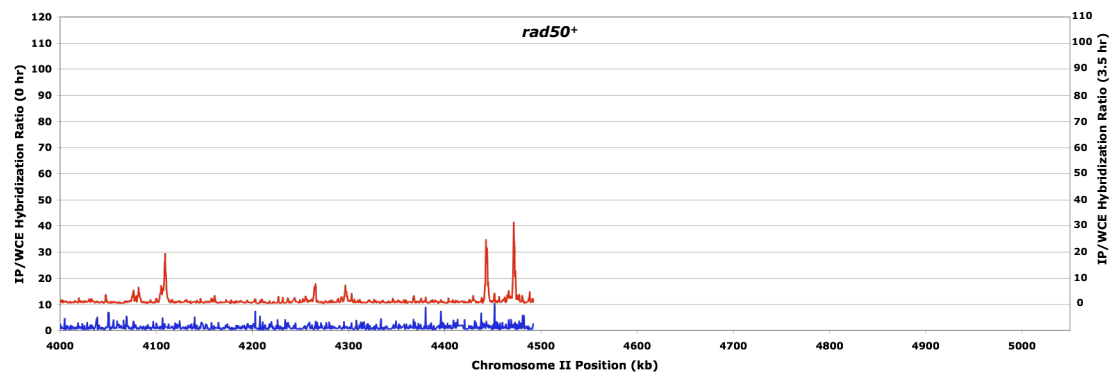

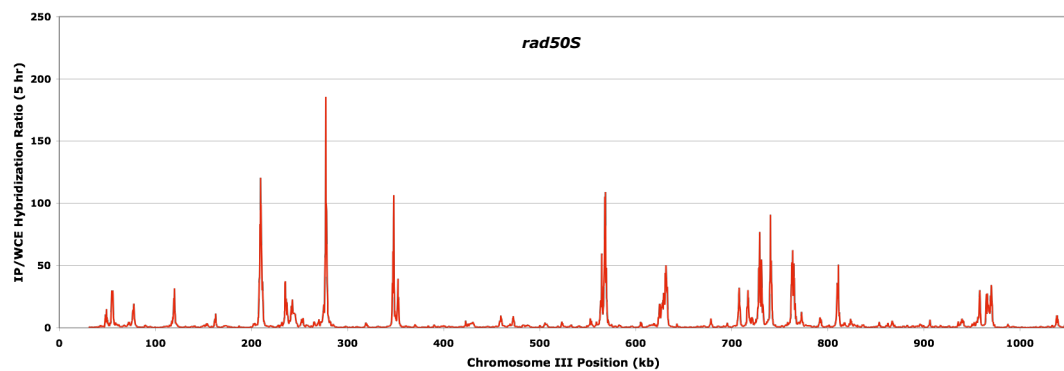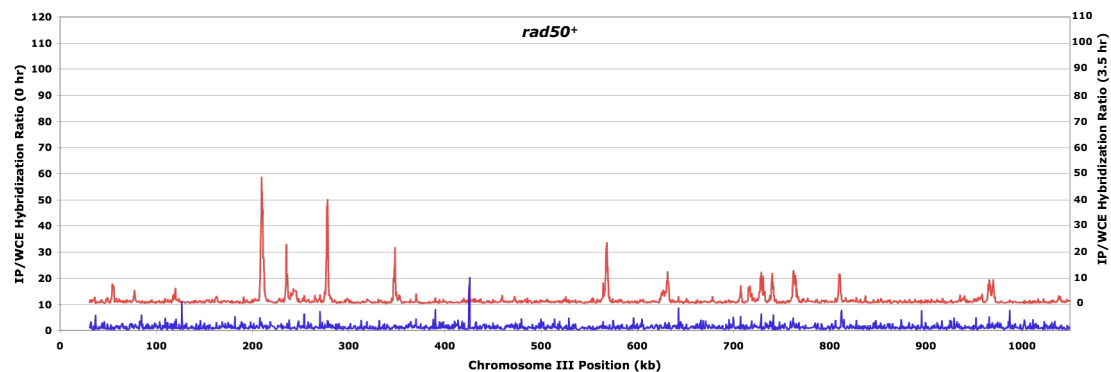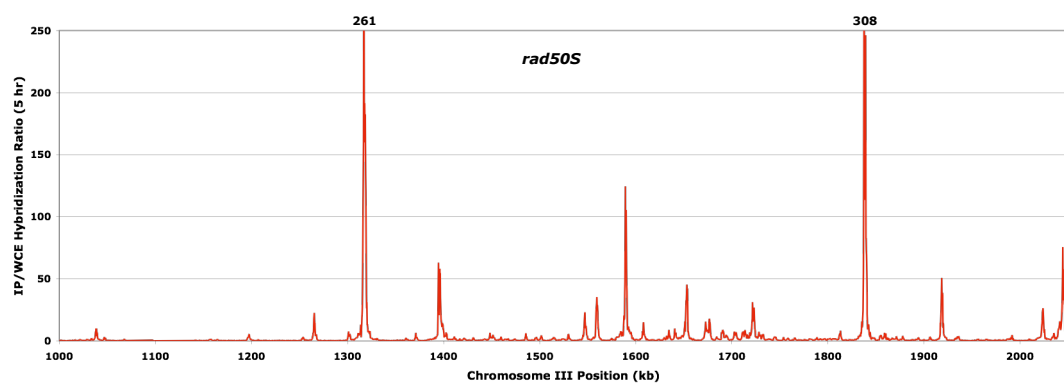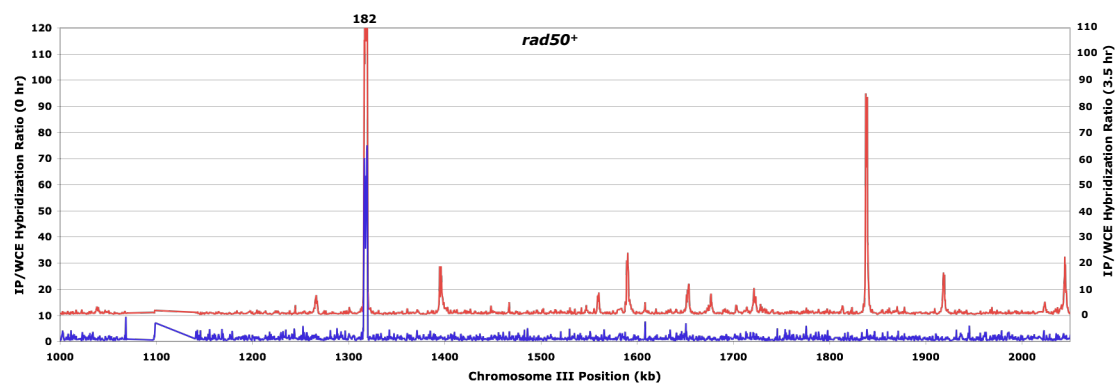

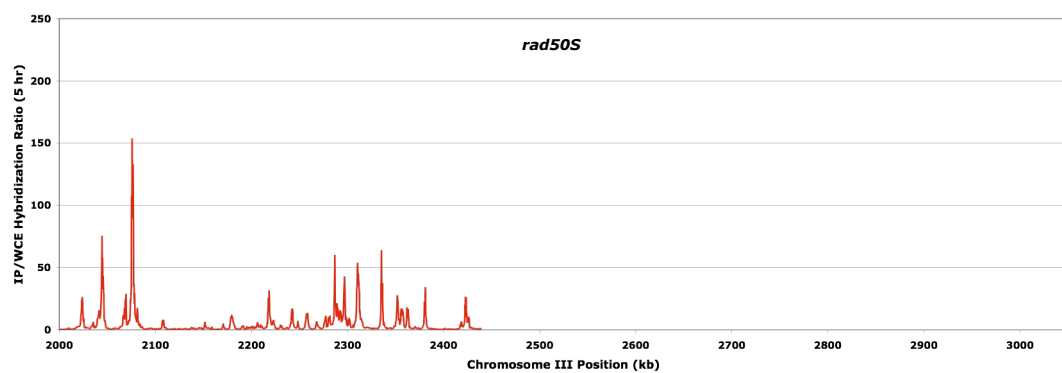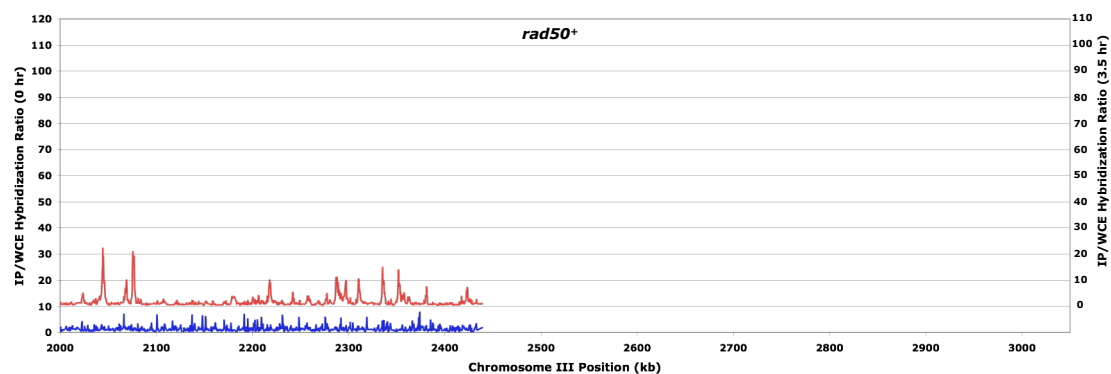

Supplement: Figure S5 — Rec12-DNA Linkages across the Entire S. pombe Genome. Shown are the median-normalized IP/WCE hybridization ratios from experiment 2 (Dataset S2). Data from induced cells (rad50+ strain GP6232 at 3.5 h after meiotic induction and rad50S strain GP6203 at 5 h) are in red. Data from uninduced (0 h) cells (rad50+ strain GP6232) are in blue. Where peaks go off-scale, the peak maximum is indicated. The data are neither smoothed nor filtered for spurious values, except for removal of 25 data points for ∼10.7 kb of DNA deleted in the rad50S strain GP6203 between direct repeats at bp 2929282–2931720 and 2939711–2942292 on chromosome I (Accession: NC_003424.3) (unpublished data). These ∼2.5 kb repeats have identities at both ends but an ∼150 bp internal region of non-homology. These 25 data points have spuriously low hybridization values for DNA from the WCE, as expected for a deletion. The strong peak seen in the 0 h data for chromosome III occurs at the site of the ade6-3049 break hotspot. It is not clear why this peak is present in the 0 hr data. It is absent from the 0 hr experiments in Dataset S1. (1.9 MB PDF) [file pgen.1000267.s005.pdf]

A

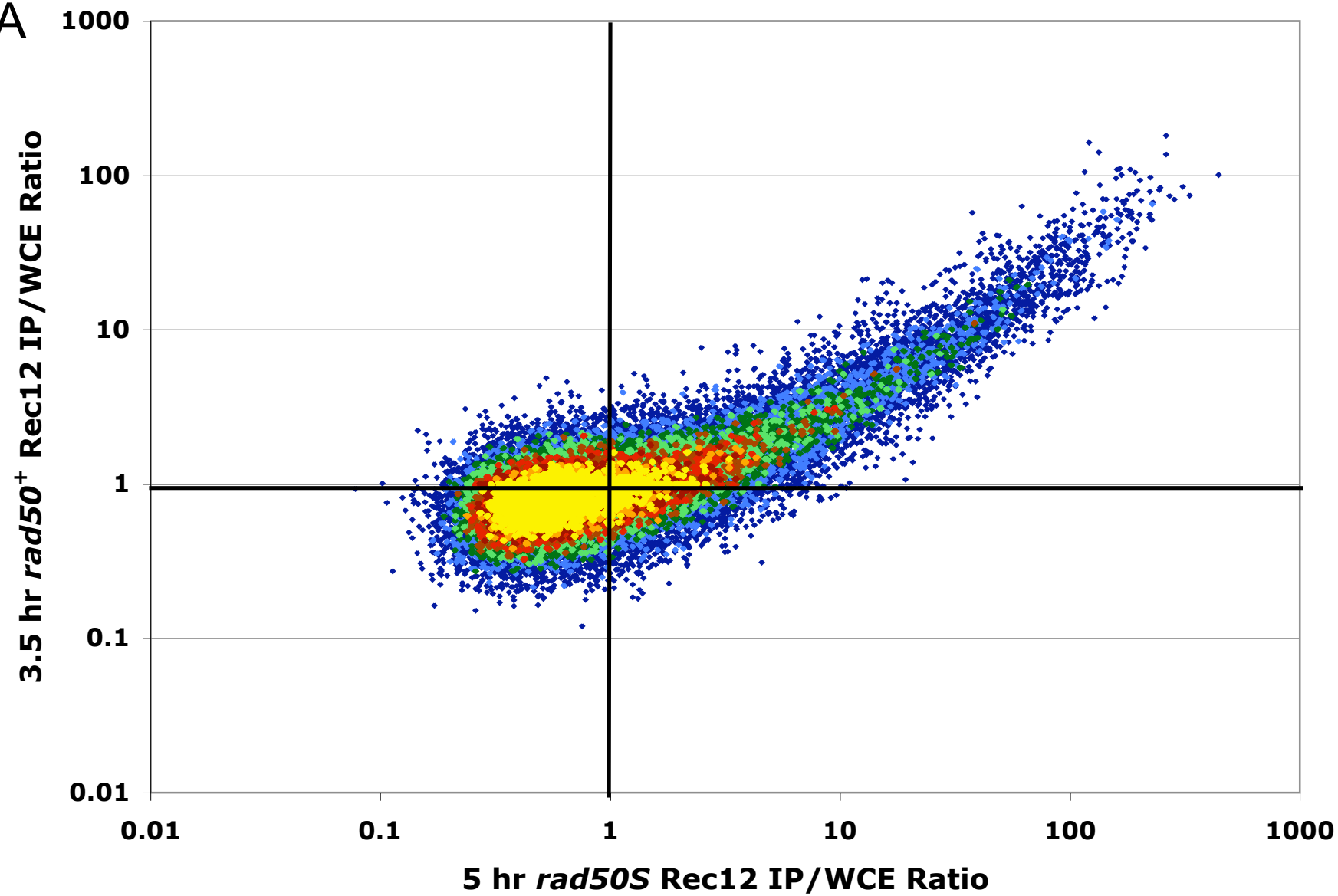

**B**

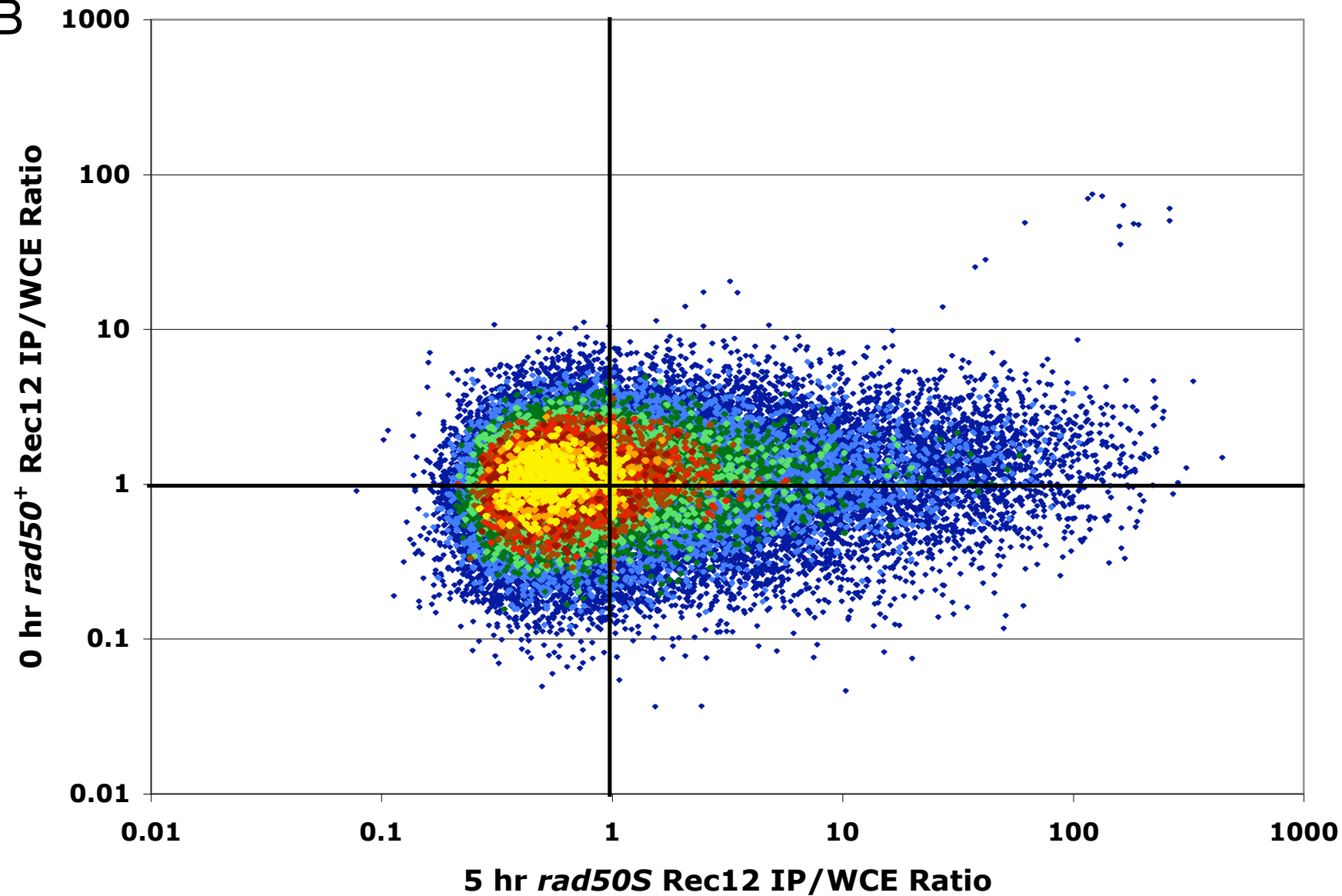

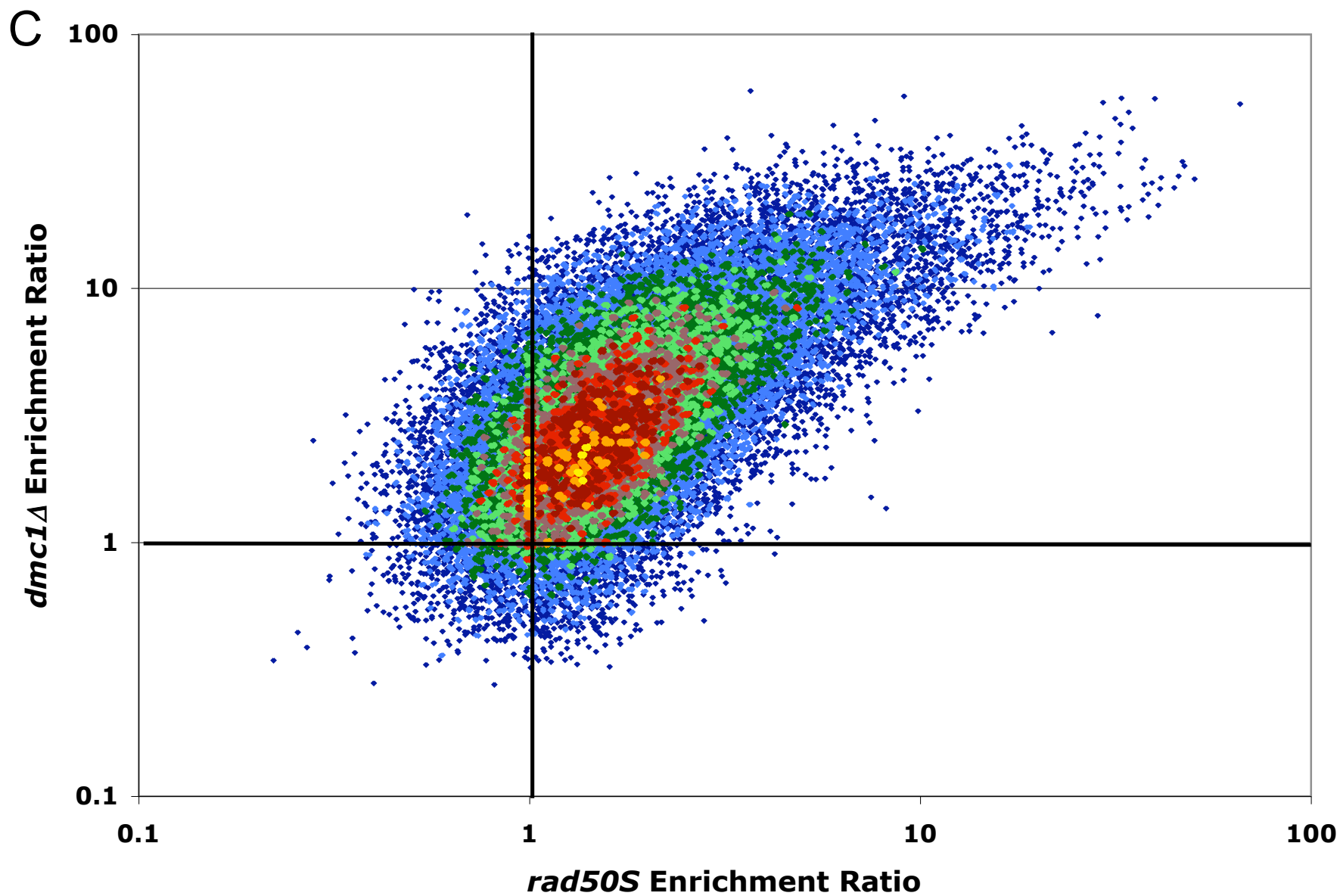

D

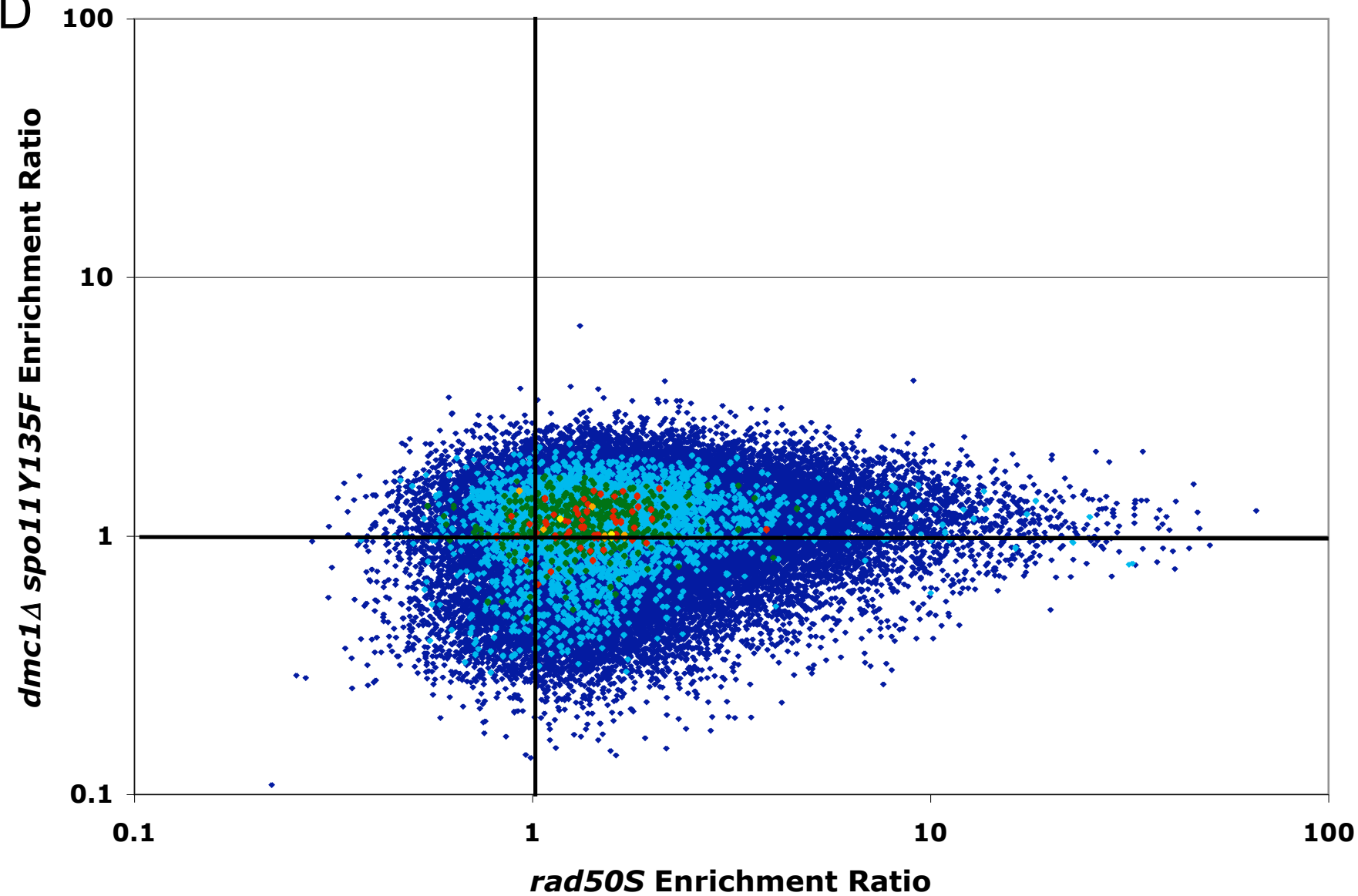

Supplement: Figure S6 — All DSB Hotspots Detected in rad50+ Are Also DSB Hotspots in rad50S; In S. cerevisiae Microarray Experiments Many Probes Show Greater Meiotic DSB Hotspot Activity in dmc1Δ Mutants Than in rad50S Mutants. The IP/WCE ratio of each probe in the rad50+ microarray hybridization is plotted against the IP/WCE ratio of the same probe in the 5 h rad50S microarray hybridization. The plots are on a log scale. Color indicates density of plotted points with yellow highest and dark blue lowest, calculated by superimposing a grid with spacing 10 0.01 on the chart (log10 enrichment values) and coloring all points within each grid square based on the number of points in that square. (A) The 3.5 h rad50+ IP/WCR ratios are positively correlated with those of the 5 h rad50S data. All probes enriched by IP of the rad50+ DNA are enriched in the rad50S DNA and vice versa. (B) The uninduced (0 h) rad50+ IP/WCR ratios show no correlation with those of rad50S, as expected for uninduced background signals. These data are from experiment 1 (Dataset S1); similar results were obtained in an independent induction of each strain (Figure 5). The number of points per grid square ranged from 1 to 37 in A and 1 to 19 in B. For comparison with S. cerevisiae, we replotted data from [8]. The normalized DSB enrichment ratio of each probe in the meiotic dmc1Δ microarray hybridization (C) or meiotic dmc1Δ spo11-Y135F (inactive Spo11) microarray hybridization (D) is plotted against the enrichment ratio of the same probe in the meiotic rad50S microarray hybridization. The plots are on a log scale. Some probes show similar enrichment in the dmc1Δ and rad50S datasets, while others are much more highly enriched in the dmcΔ dataset (C). No probes enriched in the rad50S dataset show significant enrichment in the negative control dmc1Δ spo11-Y135F dataset (D). Color indicates density of plotted points as above. The number of points per grid square ranged from 1 to 22 in C and 1 to 6 in D. (2.7 MB PDF) [file pgen.1000267.s006.pdf]

A

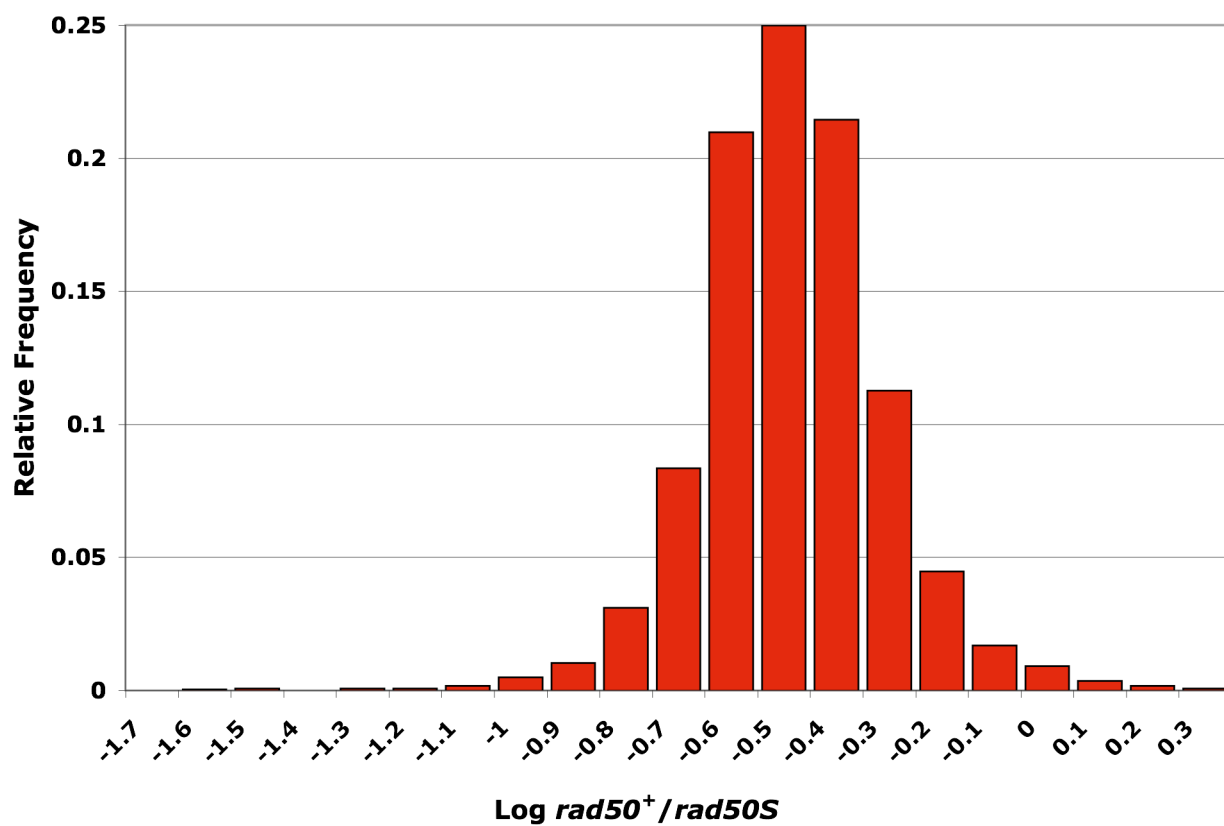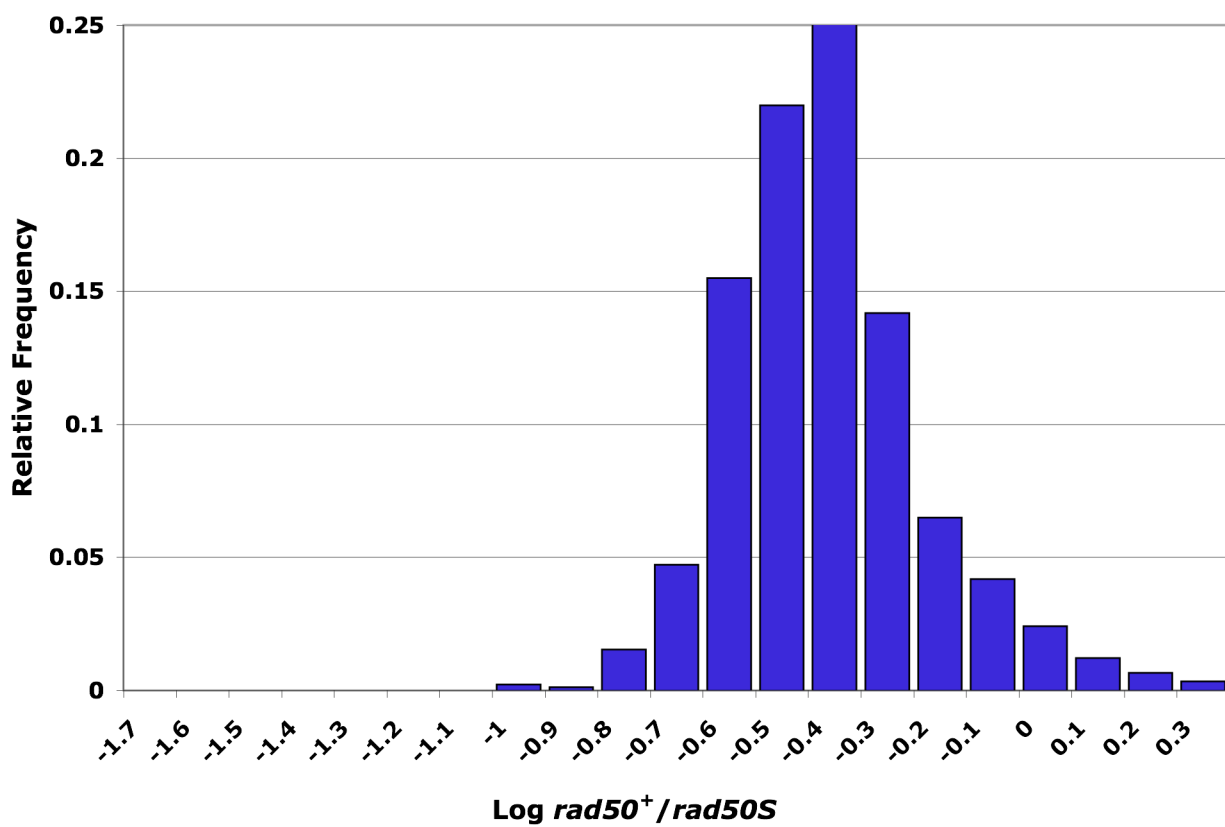

B

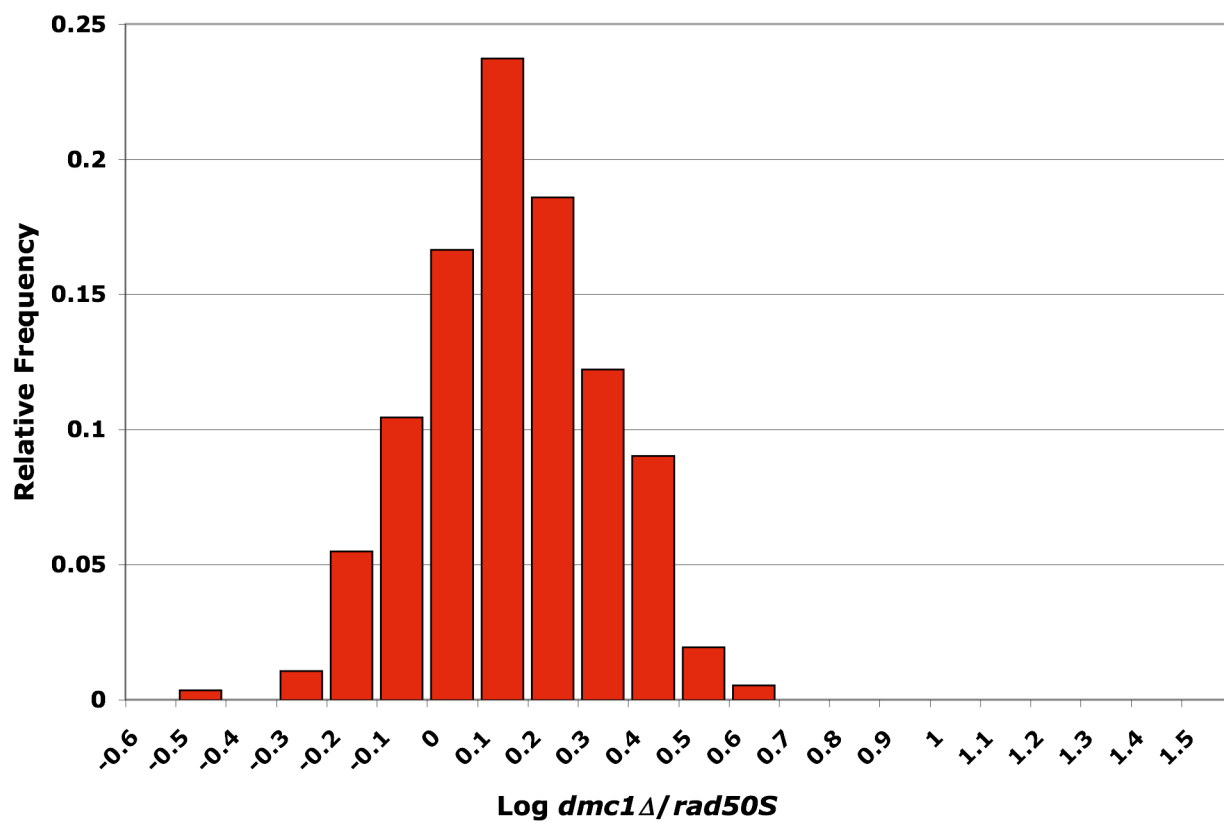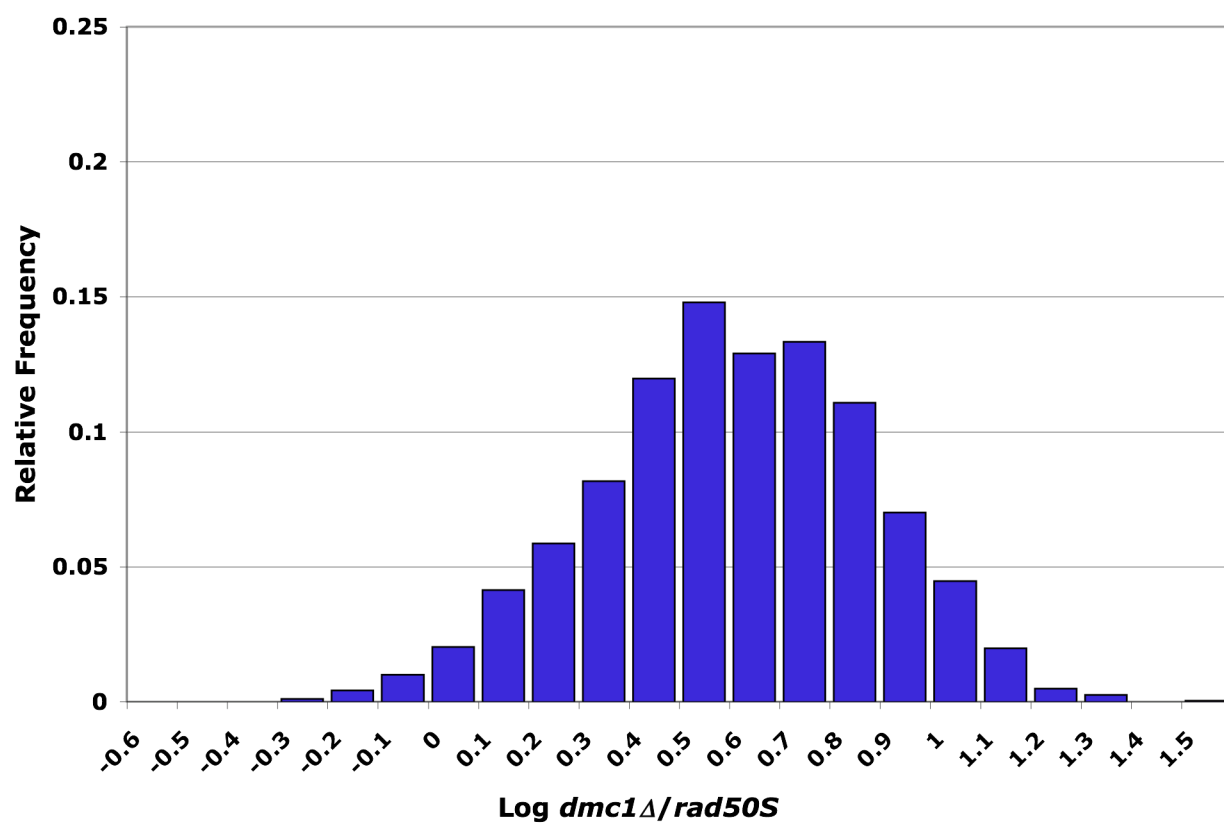

Supplement: Figure S7 — Enriched Probes Are Consistently ∼3-Fold More Highly Enriched in the 5 h rad50S Datasets of S. pombe Compared to the 3.5 h rad50+ Datasets; In Contrast, Many Probes Show Enrichment Only in the Meiotic dmc1Δ Datasets and Not the rad50S Datasets of S. cerevisiae. (A) A frequency histogram of the log10 [(3.5 h rad50+ value)/(5 h rad50S value)] for probes showing enrichment (Rec12 IP/WCE≥10) in either the S. pombe 5 h rad50S (top) or the 3.5 h rad50+ (bottom) conditions from Dataset S2 is shown. In both cases, the IP/WCE ratios are consistently ∼3-fold higher in the rad50S condition (average log10 ratio between conditions of ∼−0.5). A similar result was obtained using Dataset S1 (data not shown). (B) A frequency histogram of the log10 [(dmc1Δ value)/(rad50S value)] for probes showing enrichment (enrichment ratio≥10) in either the rad50S (top) or the dmc1Δ (bottom) conditions from [8] is shown. For probes showing enrichment in the rad50S condition, enrichment ratios are similar in the rad50S and dmc1Δ conditions (average log10 ratio between conditions of ∼0). In contrast, for many probes showing enrichment in the dmc1Δ condition, the rad50S enrichment ratio is much lower, giving significantly higher average log ratios between conditions. (0.1 MB PDF) [file pgen.1000267.s007.pdf]

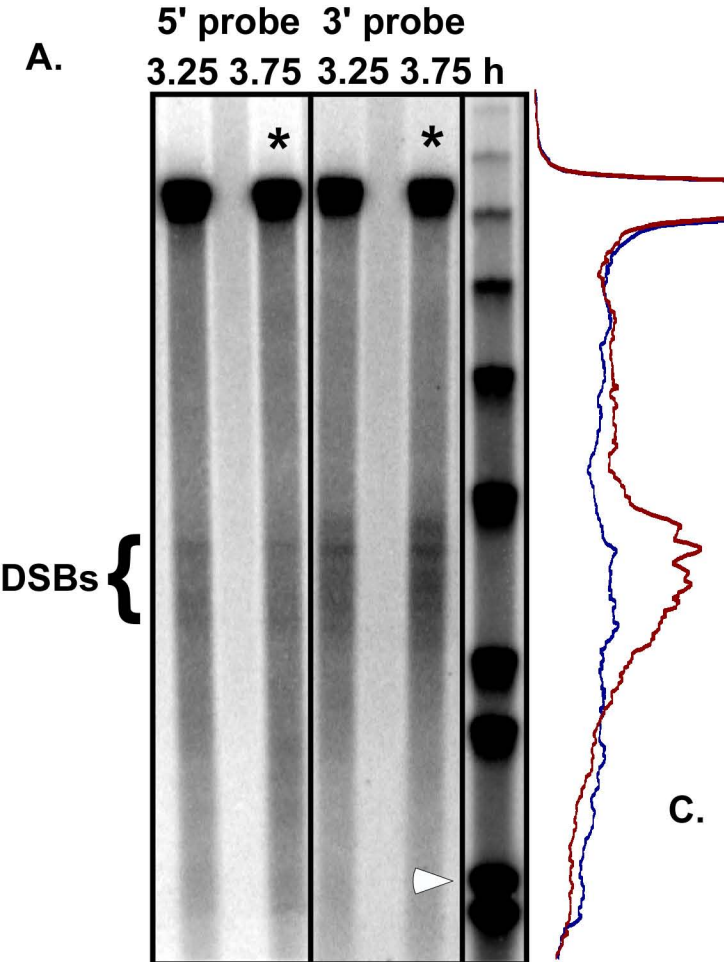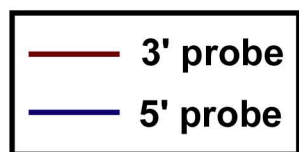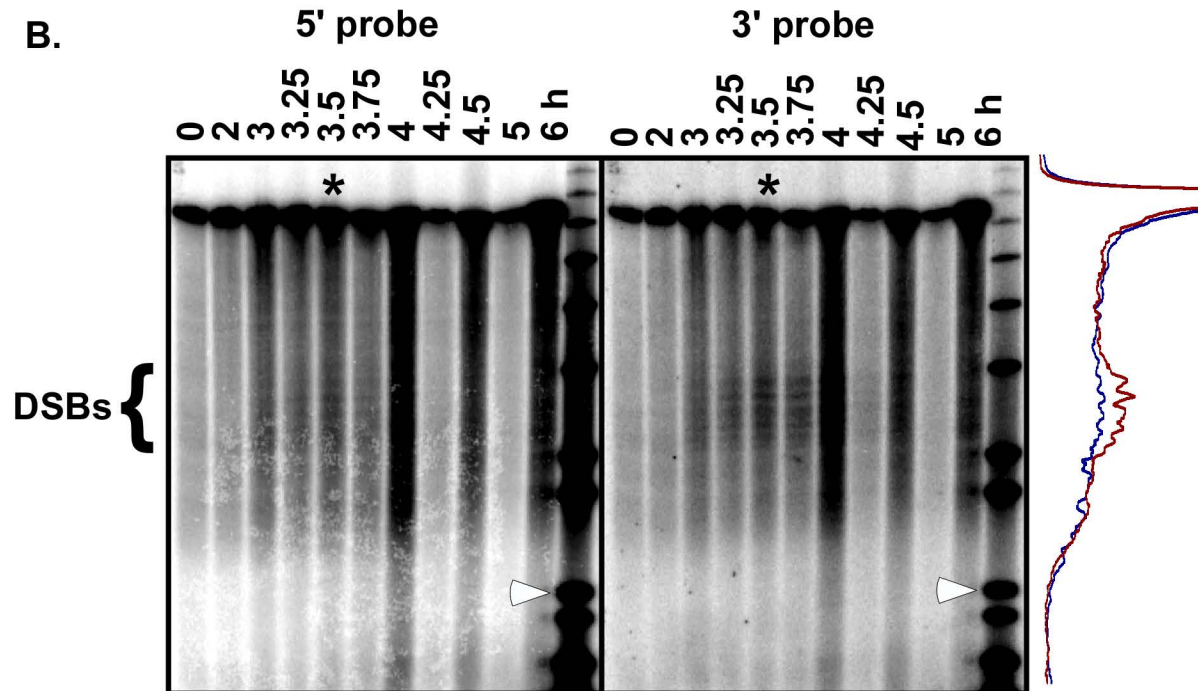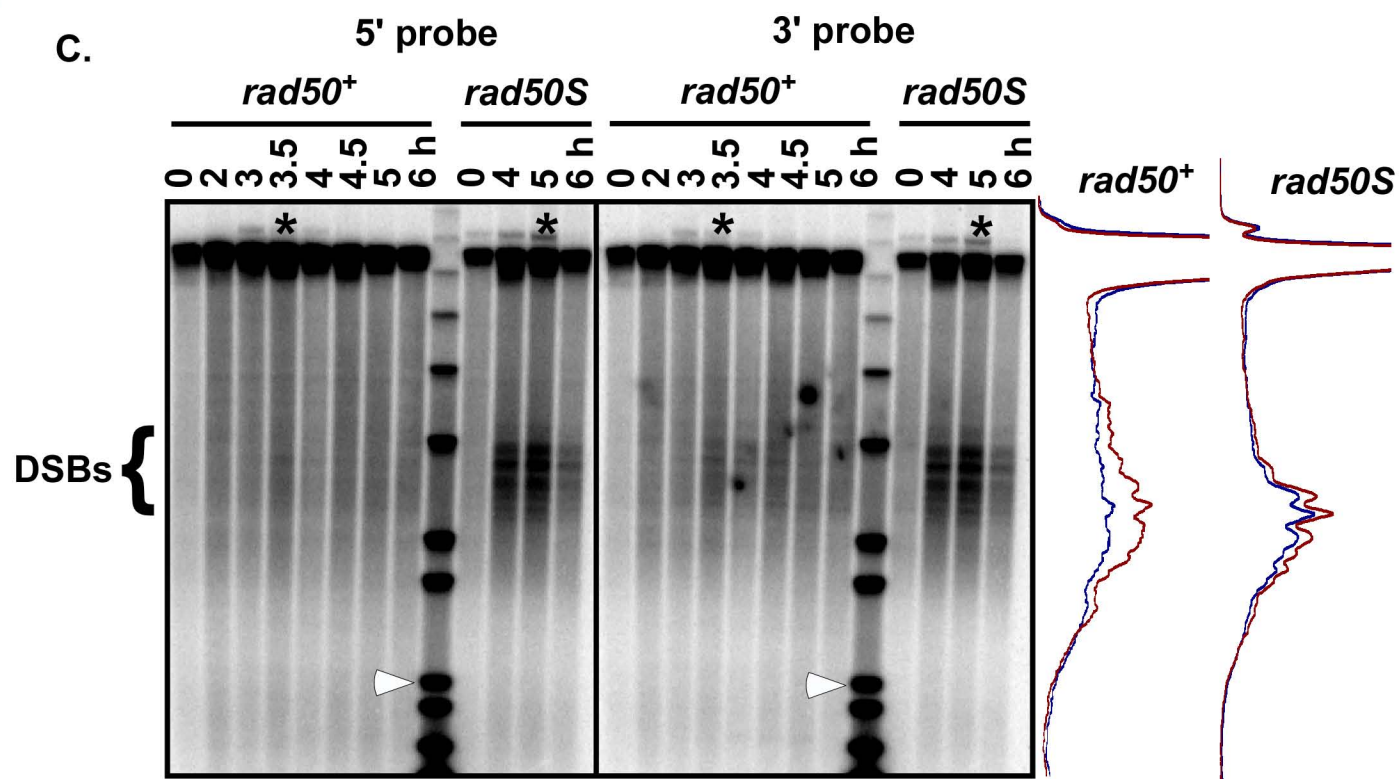

Supplement: Figure S8 — Full-length DNA Strands with 5′ and 3′ Ends at DSBs Appear in rad50+ Meiosis. DNA from meiotically induced cells with the ade6-3049 hotspot was treated with Proteinase K, digested with AflII, electrophoresed through an alkaline agarose gel (50 mM NaOH, 1 mM EDTA), and analyzed by Southern blot hybridization using two probes for the right end of the 6.6 kb AflII fragment containing ade6. Each probe was specific for either the strand with 3′-ends or the strand with 5′-ends at the DSBs as indicated. DNA from three independent experiments using strains GP6232 (rad50+) and GP3718 (rad50S) harvested at the indicated time after meiotic induction was analyzed in panels A, B, and C. [32P]-labelled 1 kb Plus DNA markers (Invitrogen) were run on the gels; white arrowheads indicate the 1 kb fragment. Line traces from phosphorimage analysis are from the times and strains indicated with an asterisk. Note that the peaks with 3′-strand probes (red lines) and 5′-strand probes (blue lines) nearly coincide, indicating that the complementary strands have indistinguishable end points at the DSBs. In DNA from rad50+ DNA the peaks are sharper and more intense with 3′-strand probes than with 5′-strand probes, indicating that the 3′-ends undergo less resection than 5′-ends, as expected from ongoing DSB repair in rad50+ strains. (0.4 MB PDF) [file pgen.1000267.s008.pdf]

A

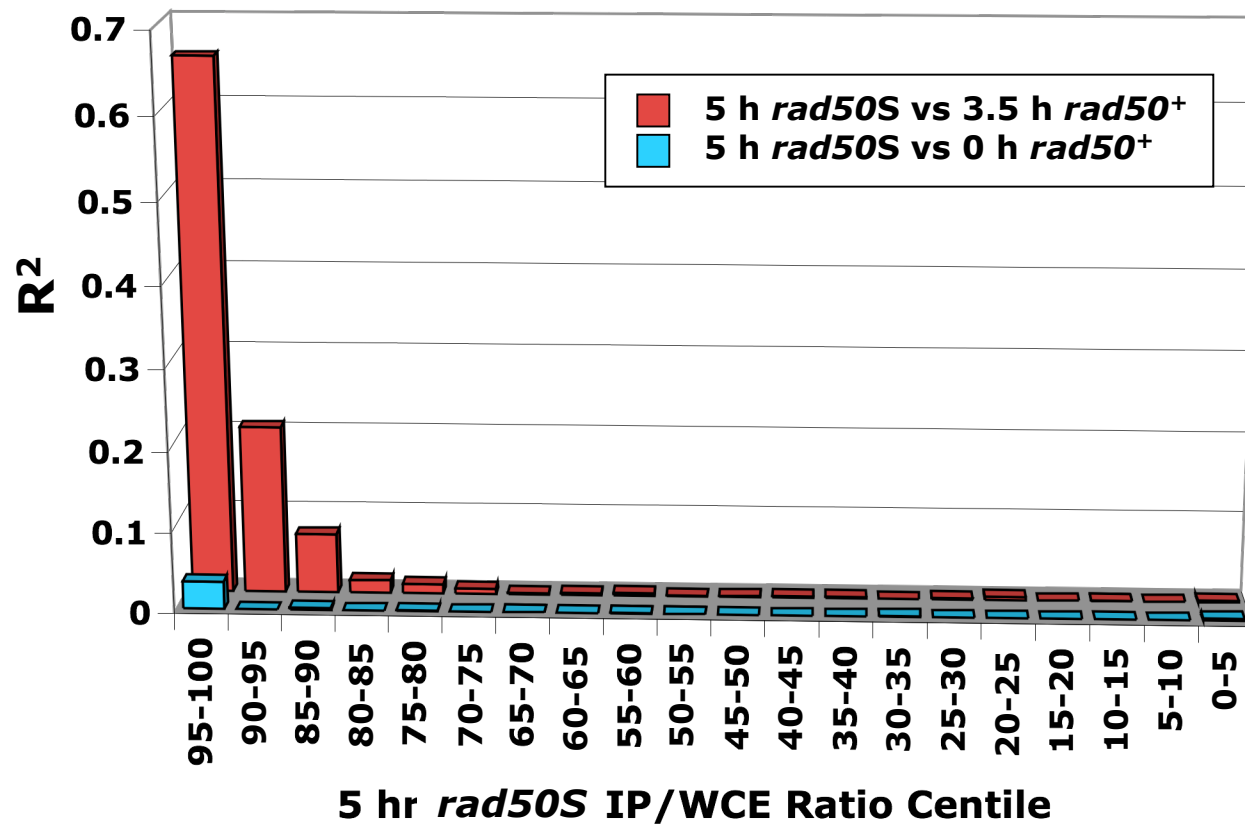

B

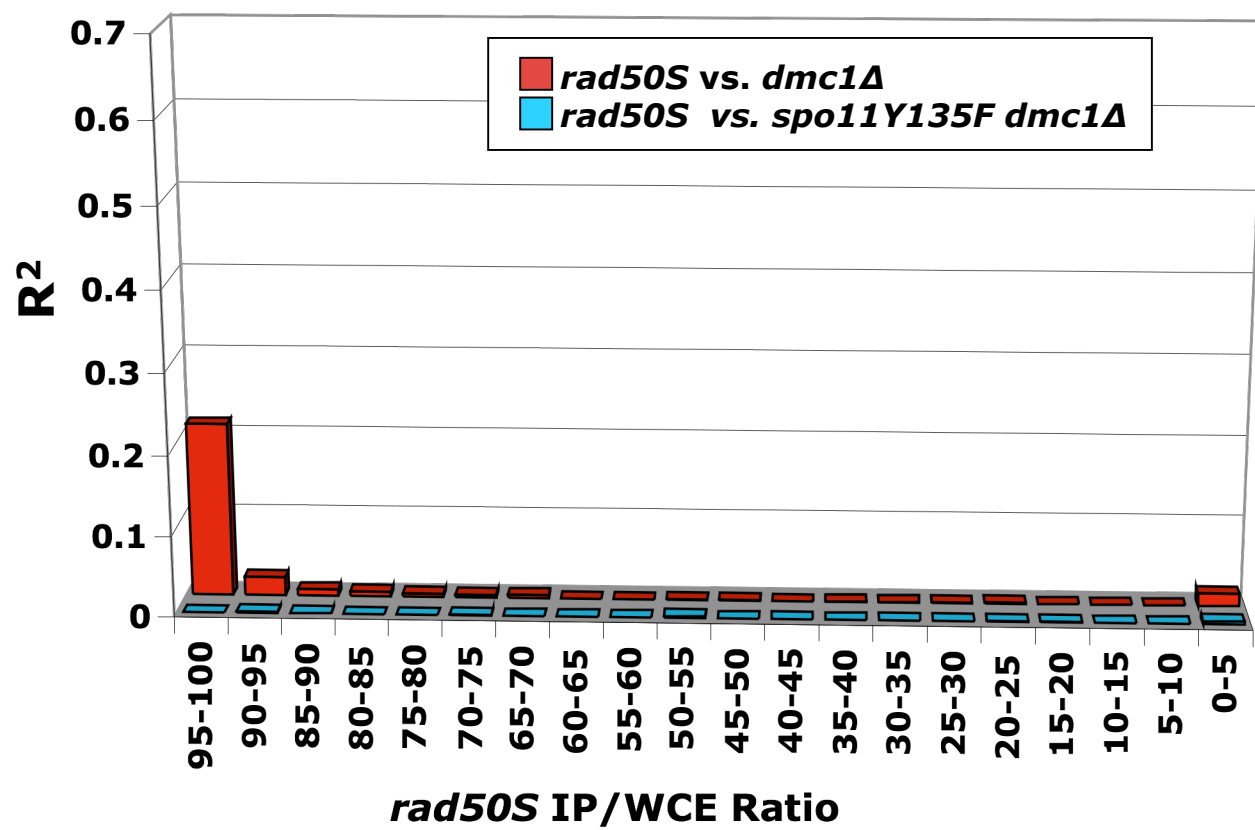

Supplement: Figure S9 — IP/WCE Ratios for Rec12-Enriched Probes Are Highly Correlated between the 3.5 h rad50+ and 5 h rad50S Data; A Much Weaker Correlation Is Seen between the Meiotic DSB Enrichment Ratios of dmc1Δ and rad50S Mutants of S. cerevisiae. (A) Probes were ranked by the 5 h rad50S IP/WCE ratio from S. pombe Dataset S2 and divided into 20 groups of equal numbers of probes. For each group of probes the Pearson product-moment correlation coefficient (R) was calculated first between the log 5 h rad50S and log 3.5 h rad50+ ratio values and then between the log 5 h rad50S and log 0 h rad50+ ratio values. R2 values, representing the association between the paired ratios, are plotted against the ordered five-centile groupings (where 100 is the 5 h rad50S centile with the highest IP/WCE values). (B) Probes were ranked by the meiotic rad50S IP/WCE ratio from S. cerevisiae, taken from [8], and divided into 20 groups of equal numbers of probes. For each group of probes the Pearson product-moment correlation coefficient (R) was calculated first between the log meiotic rad50S and log meiotic dmc1Δ ratio values and then between the log meiotic rad50S and log meiotic dmc1Δ spo11-Y135F (negative control) values. R2 values, representing the association between the paired ratios, are plotted against the ordered five-centile groupings (where 100 is the meiotic rad50S centile with the highest IP/WCE values). (0.3 MB PDF) [file pgen.1000267.s009.pdf]

**A**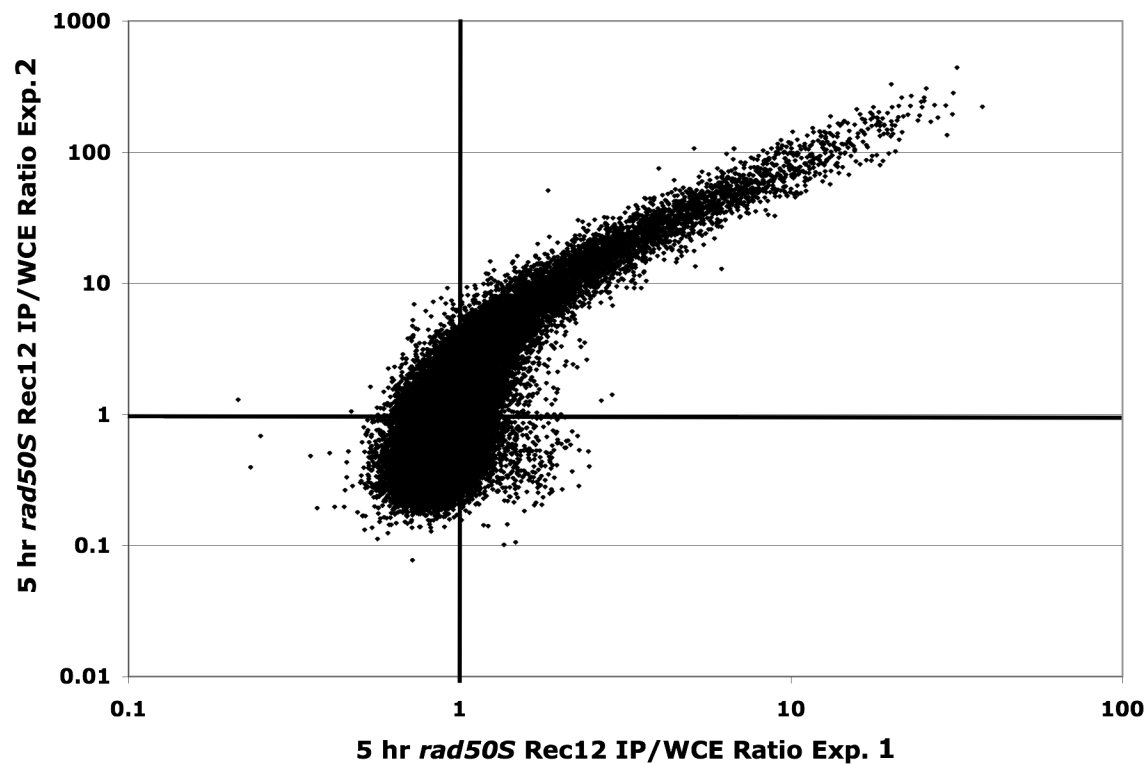**B**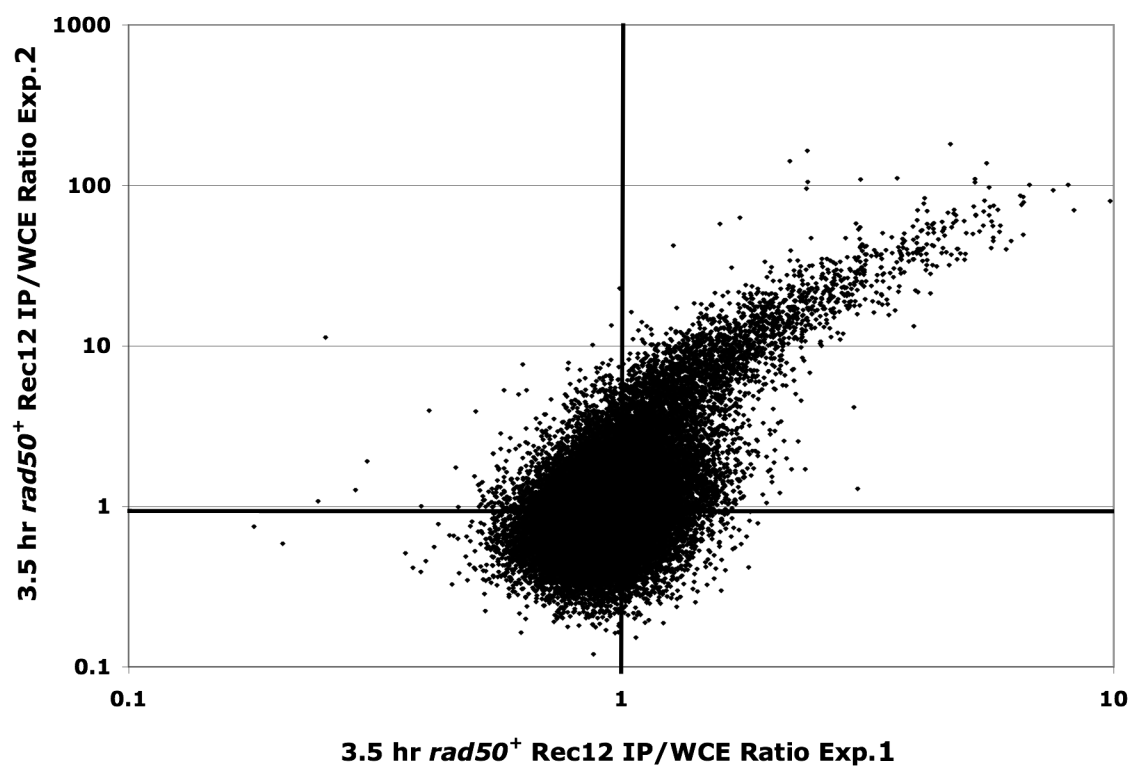

Supplement: Figure S10 — A Consistent Subset of Probes Shows Rec12 IP/WCE Enrichment across Hybridizations in Both rad50+ and rad50S Backgrounds. (A) The 5 h Rec12 IP/WCE ratio of each probe in the second rad50S microarray hybridization (Dataset S2) is plotted against the 5 h IP/WCE ratio of the same probe in the first rad50S microarray hybridization (Dataset S1). (B) The 3.5 h Rec12 IP/WCE ratio of each probe in the second rad50+ microarray hybridization (Dataset S2) is plotted against the 3.5 h IP/WCE ratio of the same probe in the first rad50+ microarray hybridization (Dataset S1). In both (A) and (B), essentially all probes showing enrichment in one hybridization also show enrichment in the other. The plots are on a log scale. (0.1 MB PDF) [file pgen.1000267.s010.pdf]
